# Fra-1 promotes gastric cancer progression by regulating macrophage polarization and transcriptionally activating HMGA2 expression

**DOI:** 10.1038/s41420-025-02724-1

**Published:** 2025-10-06

**Authors:** Feng Zeng, Jiaying Cao, Shan Liao, Yan Chen, Qian He, Yan Lei, Juan Xu, Yanhong Zhou

**Affiliations:** 1 Department of Critical Care Medicine, The Affiliated Cancer Hospital of Xiangya School of Medicine Central South University/Hunan Cancer Hospital, Changsha, Hunan China; 2 Cancer Research Institute, Basic School of Medicine, Central South University, Changsha, Hunan China; 3 Department of Pathology, The Third Xiangya Hospital of Central South University, Changsha, Hunan China; 4 Department of Radiation Oncology, The Affiliated Cancer Hospital of Xiangya School of Medicine, Central South University/Hunan Cancer Hospital, Changsha, Hunan China; 5 Department of Blood Transfusion, The Affiliated Cancer Hospital of Xiangya School of Medicine, Central South University/Hunan Cancer Hospital, Changsha, Hunan China

**Keywords:** Cancer, Cell biology

## Abstract

Gastric cancer is a common malignant tumour of gastrointestinal tract with high incidence and low early diagnosis rate. Surgery is its main treatment modality, but some patients have poor prognosis. The rise of immunotherapy provides a new therapeutic strategy for gastric cancer treatment. Elucidating the mechanism of action of immune cells in the tumour microenvironment is the cornerstone for developing new tumour immunotherapy strategies. Previous studies have found that Fra-1 is highly expressed in gastric cancer and is closely associated with macrophage polarisation. In order to further elucidate the specific mechanism, this study firstly used in vitro co-culture experiments to verify that the high expression of Fra-1 in gastric cancer cells induced macrophage M2 polarisation; then, whole proteomics combined with in vitro cellular experiments were used to clarify the specific mechanism by which Fra-1 induced macrophage M2 polarisation by regulating HMGA2 expression in gastric cancer cells. Finally, in vivo experiments further elucidated that Fra-1 induces macrophage polarisation in gastric cancer cells and participates in tumourigenesis and development. The aim of this study was to systematically elucidate the role of Fra-1 in the tumour microenvironment and its possible mechanisms, and to provide an experimental basis for the development of immunotherapeutic strategies for gastric cancer.

## Introduction

Gastric cancer (GC), originating from the gastric mucosal epithelium, is the fifth most common malignant tumor and the fourth leading cause of cancer-related deaths globally [1]. This highly heterogeneous disease is primarily treated with endoscopic resection for early-stage cases, while advanced cases often require continuous chemotherapy. The first-line therapy typically involves a combination of platinum and fluorouracil. Despite these treatments, patients often face short survival periods and limited efficacy. Current targeted therapies include trastuzumab for first-line treatment of HER2-positive patients, ramucirumab for second-line anti-angiogenic treatment, and nivolumab or pembrolizumab for third-line anti-PD-1 treatment [2]. Although advancements in early diagnosis and adjuvant therapy have improved gastric cancer prognosis, radical surgery remains the primary treatment. Unfortunately, gastric cancer can recur with local or distant metastasis after radical gastrectomy, leading to a less than ideal prognosis. The development of gastric cancer is a complex, multi-step process influenced by various factors, including microorganisms such as Helicobacter pylori [3, 4], oncogenes like Fra-1, BRD4, and CD90 [5–7], tumor suppressor genes, and immune factors [3, 8]. The underlying mechanisms of gastric cancer are not yet fully understood, necessitating further research to uncover its pathogenesis and identify potential molecular targets for early diagnosis and prognosis prediction.

Fos associated antigen-1 (Fra-1), a member of the activator protein-1 (AP-1) transcription factor superfamily and the Fos family, shares a high degree of homology with c-FOS in its basic domain [9, 10]. As a crucial nuclear transcription factor, Fra-1 promotes cancer cell proliferation, differentiation, and apoptosis. It is often abnormally expressed in various cancer cells and tumor tissues, playing a significant role in tumorigenesis and tumor progression [11, 12]. Fra-1 regulates the expression of epithelial-mesenchymal transition (EMT) related factors, thereby promoting EMT, invasion, and carcinogenesis, which are key steps in tumor progression [13–15]. Furthermore, Fra-1 significantly influences the expression of cell cycle proteins, contributing to tumor progression. In our previous research, we discovered that Fra-1 is highly expressed in gastric cancer tissues. It inhibits gastric cancer cell apoptosis, increases the proportion of cells in the S phase, and stimulates cell proliferation by modulating the PI3K/AKT signaling pathway [5]. Despite these findings, the precise role and underlying mechanisms of Fra-1 in gastric cancer development warrant further investigation.

The tumor microenvironment (TME) is a complex ecosystem that tumors depend on for survival and growth, comprising tumor cells, stromal cells, and various immune cells, as well as their secreted products like cytokines and chemokines [16–18]. Within this environment, tumor-associated macrophages (TAMs) play a crucial role in tumor progression through multiple pathways [19]. Macrophages are categorized into two main types based on their activation and function: classically activated macrophages (M1 type) and alternatively activated macrophages (M2 type) [20, 21]. Early in tumor development, TAMs often display an M1 phenotype, which shifts to a predominantly M2 phenotype as the tumor progresses [22–29]. M1-type TAMs activate anti-tumor immunity, producing nitric oxide (NO), reactive oxygen species (ROS), and pro-inflammatory cytokines such as TNF-α, IL-1β, and IL-6. Conversely, M2-type TAMs, which are pro-tumorigenic, secrete immunosuppressive cytokines like transforming growth factor beta (TGF-β) and interleukins (IL-10, IL-13). These cytokines facilitate tumor progression by recruiting Treg cells and suppressing the effector functions of CD4+ and CD8 + T cells within the TME [30, 31]. Additionally, macrophages contribute to tumor progression by inducing angiogenesis, primarily through the VEGF family, which includes PlGF and VEGF-A/B/C/D. These growth factors stimulate angiogenesis by binding to receptor tyrosine kinase receptors (TKRs), such as VEGFR-1/2/3 [32–34], thereby promoting tumor growth [35, 36]. As our understanding of tumor immunology deepens, new therapeutic strategies are emerging, such as clearing macrophages or modifying their phenotype. Given the complexity of malignant tumor progression, combination therapies may enhance anti-tumor responses and therapeutic outcomes. Therefore, elucidating the role of Fra-1 in macrophage polarization and its association with gastric cancer progression could uncover novel therapeutic targets.

In this study, we systematically investigated the role of Fra-1 in gastric cancer progression. Initially, we established the impact of Fra-1 on the disease’s progression. We then identified HMGA2 as a key downstream protein regulated by Fra-1 and demonstrated that Fra-1 enhances gastric cancer progression through its effects on HMGA2. Utilizing ChIP-qPCR technology and dual luciferase reporter gene assays, we confirmed that Fra-1 transcriptionally regulates HMGA2 by binding to its promoter region. Additionally, we verified that HMGA2 influences the polarization and functionality of macrophages. Further recovery experiments substantiated that Fra-1 promotes cancer progression by transcriptionally activating HMGA2, which promotes the secretion of CCL2 from tumor cells, which in turn induces macrophage polarization and vascular endothelial growth factor (VEGF) secretion, leading to tumor angiogenesis. Finally, in vivo experiments confirmed Fra-1’s role in modulating gastric cancer disease progression. Our findings significantly contribute to the understanding of Fra-1’s role and mechanisms in gastric cancer pathogenesis and progression. They also hold promise for early diagnosis, identification of potential therapeutic targets, treatment of late-stage metastasis, and prognosis prediction in gastric cancer.

## Results

### Fra-1 is highly expressed in gastric cancer tissue, promoting gastric cancer progression

**Fig. 1:**
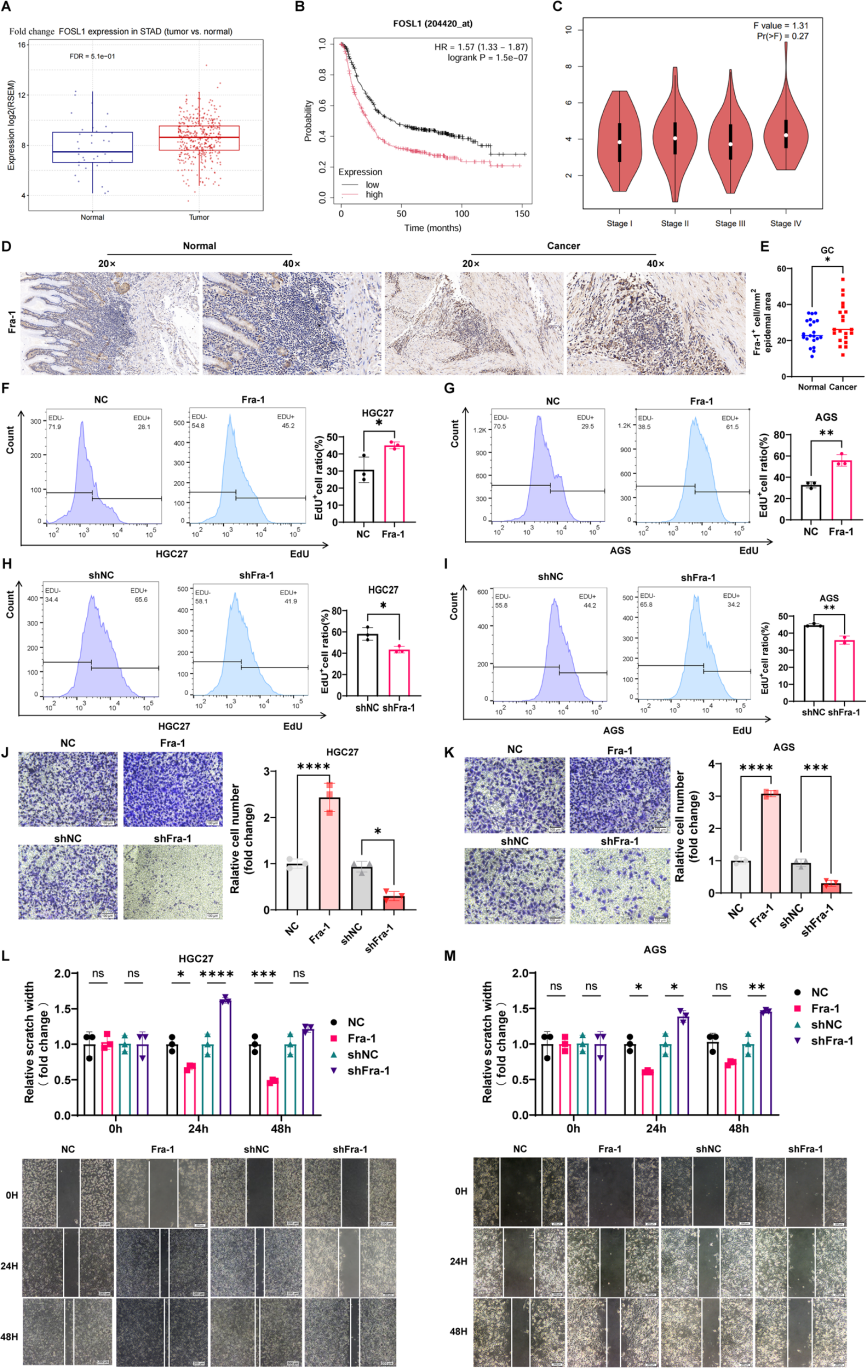
High expression of Fra-1 in gastric cancer correlates with poor prognosis and enhanced cancer cell properties. **A** Box plot showing the expression levels of Fra-1 in the gastric cancer cohort (STAD) from the TCGA database. “Normal” refers to gastric glandular epithelium, while “Tumor” refers to gastric cancer tissue. FDR (False Discovery Rate) = 5.1e-01. **B** Kaplan–Meier overall survival curve analysis based on Fra-1 expression levels in gastric cancer patients from the GEO dataset. The graph illustrates the survival rates of patients with high Fra-1 expression versus those with low Fra-1 expression at various time points. Hazard Ratio (HR) = 1.57 (95% CI: 1.33–1.87), log-rank p-value = 1.5e-07. **C** GEPIA2 database analysis of Fra-1 expression levels across different clinical stages of gastric cancer. F value = 1.31, Pr (>F) = 0.27. **D** Representative immunohistochemical images showed the expression of Fra-1 in gastric cancer tissues and corresponding paracancerous tissues. 20×: 50 μm; 40×: 20 μm. **E** Immunohistochemical semi-quantitative statistical chart. Normal: 21 cases; Cancer: 23 cases; The p-value was calculated through unpaired t-test correction. **F**, **G** Overexpression of Fra-1 in gastric cancer cells HGC27/AGS was achieved, and an EdU cell proliferation assay combined with flow cytometry was utilized to assess changes in the proliferation ability of gastric cancer cells. The p-value was calculated through unpaired t-test correction. **H**, **I** Knockdown of Fra-1 in gastric cancer cells HGC27/AGS was performed, and the effect on cell proliferation was measured using an EdU cell proliferation assay combined with flow cytometry analysis. The p-value was calculated through unpaired t-test correction. **J**, **K** The effect of Fra-1 knockdown on the invasion ability of gastric cancer cells HGC27/AGS was determined using a Transwell cell invasion assay. Scale bar, 100 μm. The p-value was calculated through unpaired t-test correction. **L**, **M** A scratch healing assay was conducted to investigate the impact of Fra-1 on the migration ability of gastric cancer cells HGC27/AGS. Scale bar, 200 μm. The p-value was calculated through unpaired t-test correction. All experiments were independently repeated three or more times, and the data presented are from representative individual experiments. “ns” indicates no significant difference; “*“ indicates *p* < 0.05; “**” indicates *p* < 0.01; “***” indicates *p* < 0.001; “****” indicates *p* < 0.0001.

In this study, we initially analyzed the TCGA database and discovered that Fra-1 is significantly overexpressed in gastric cancer tissue compared to normal gastric glandular epithelial tissue (Fig. 1A). Kaplan–Meier survival analysis revealed that patients with high Fra-1 expression had shorter overall survival times than those with low expression levels. Additionally, GEPIA2 database analysis indicated that Fra-1 expression levels vary among gastric cancer patients at different stages (Fig. 1B, C). In addition, to further validate the expression of Fra-1 in clinical samples, we collected 21 fresh gastric cancer tissue specimens and 21 corresponding paracancerous tissue specimens. Immunohistochemistry experiments revealed that Fra-1 was significantly overexpressed in gastric cancer tissues compared to paracancerous tissues (Fig. 1D, E). These findings suggest a strong association between high Fra-1 expression and poor prognosis in gastric cancer. To elucidate Fra-1’s role in gastric cancer progression, we experimentally overexpressed or knocked down Fra-1 in gastric cancer cells. The efficiency of this manipulation was confirmed through RT-qPCR, demonstrating successful overexpression or knockdown of Fra-1 (Supplementary Fig. 1A, B). These experiments laid the groundwork for further investigation into Fra-1’s contribution to the malignant behavior of gastric cancer cells.

Following the initial findings, we manipulated the expression levels of Fra-1 in the gastric cancer cell line HGC27. We overexpressed or knocked down Fra-1 and then assessed the cells’ proliferative capacity using an EdU cell proliferation assay combined with flow cytometry. The results demonstrated that the proportion of proliferating cells in the Fra-1 overexpression group was significantly higher than in the control group (*p* < 0.05). Conversely, knocking down Fra-1 led to a decrease in the proliferation rate (*p* < 0.05) Similar results were observed in the gastric cancer cell line AGS, with overexpression of Fra-1 enhancing cell proliferation and knockdown reducing it (*p* < 0.01) (Fig. 1F–I). These results collectively indicate that Fra-1 significantly promotes the proliferation of gastric cancer cells, as evidenced by increased proliferation with Fra-1 overexpression and decreased proliferation with Fra-1 knockdown.

We further investigated the role of Fra-1 in the invasiveness of gastric cancer cells by manipulating its expression in the HGC27 cell line. Utilizing a transwell invasion assay, we observed that the number of cells that penetrated the matrix gel was significantly higher in the Fra-1 overexpression group compared to the control group (*p* < 0.0001), suggesting a substantial increase in invasiveness with Fra-1 overexpression. In contrast, knocking down Fra-1 resulted in a significant reduction in the number of cells invading through the matrix gel (p < 0.05), indicating a decreased invasive ability (Fig. 1J). Consistent with the HGC27 results, the AGS cell line also showed a significant increase in invasiveness with Fra-1 overexpression (p < 0.001) and a decrease with Fra-1 knockdown (Fig. 1K). These findings indicate that Fra-1 significantly enhances the invasive capabilities of gastric cancer cells, as evidenced by the increased invasion with overexpression and the reduced invasion with knockdown.

To assess the influence of Fra-1 on the migration capabilities of gastric cancer cells, we manipulated Fra-1 expression in the HGC27/AGS cell lines and conducted scratch wound healing assays. At 24 h, the wound closure in the Fra-1 overexpression group was significantly faster than in the control group (*p* < 0.05). This difference became more pronounced at 48 h, with the overexpression group showing a markedly narrower scratch width (p < 0.001), indicating a stronger cell healing ability. Conversely, Fra-1 knockdown resulted in a slower wound closure rate, with a wider scratch width compared to the shNC group at 48 h (*p* < 0.05), indicating a weakened migration ability (Fig. 1L, M). These results demonstrate that Fra-1 overexpression significantly enhances the migration ability of gastric cancer cells, while its knockdown has the opposite effect. Collectively, our findings indicate that Fra-1 is highly expressed in gastric cancer tissue and plays a crucial role in promoting the proliferation, invasion, and migration of gastric cancer cells, which are key factors in the disease’s malignant progression.

### Fra-1 positively regulates HMGA2 and promotes gastric cancer progression

**Fig. 2:**
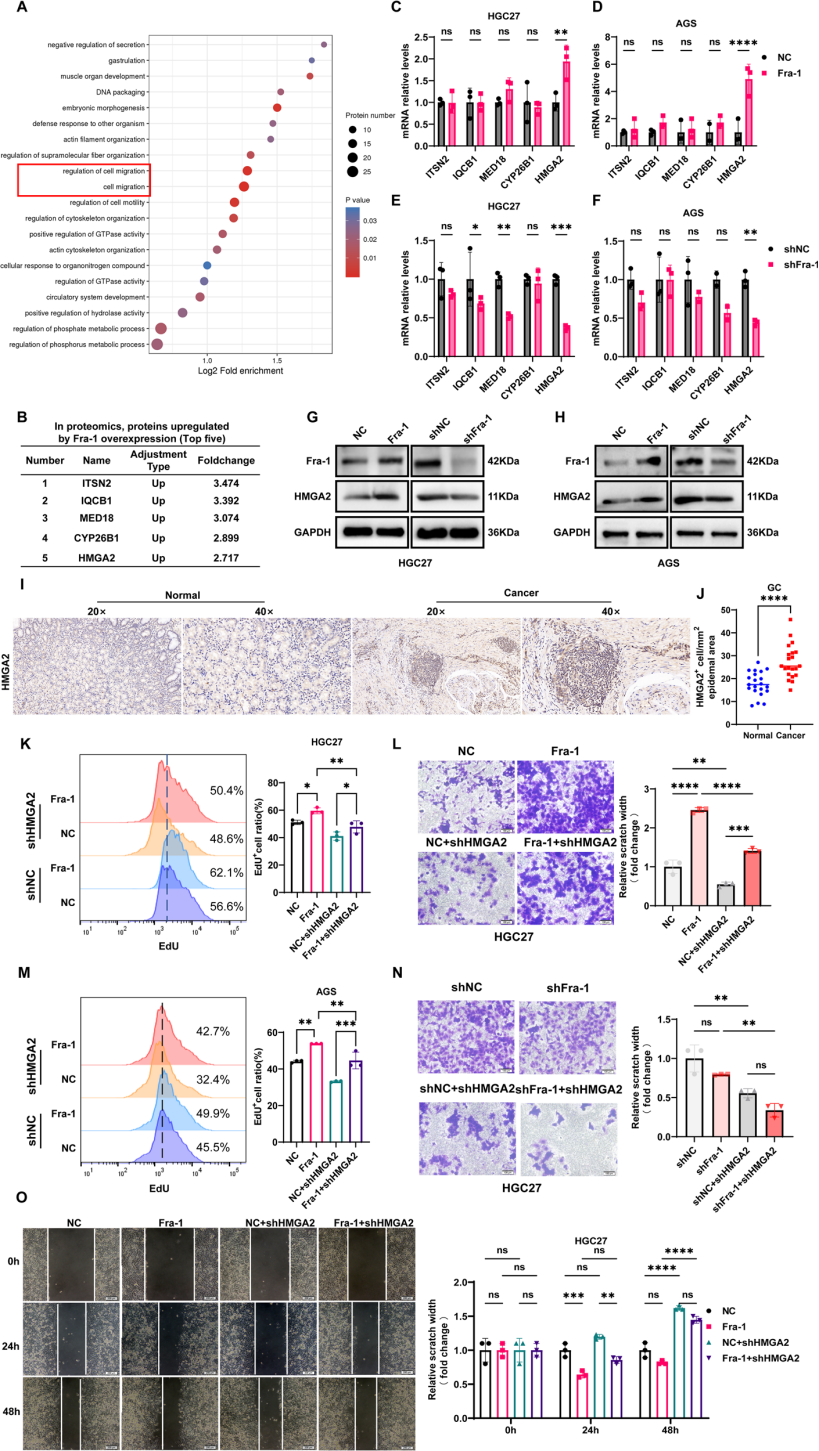
Fra-1 Positively regulates HMGA2 to promote gastric cancer progression. **A** Gene Ontology (GO) enrichment analysis of differentially expressed genes identified in the proteomic profiling of gastric cancer cells overexpressing Fra-1. **B** The top five upregulated genes from the proteomic analysis of gastric cancer cells with Fra-1 overexpression. **C**–**F** RT-qPCR experiments were conducted to assess the impact of Fra-1 overexpression and knockdown on the mRNA levels of the top five upregulated proteins: ITSN2, IQCB1, MED18, CYP26B1, and HMGA2 in gastric cancer cells HGC27/AGS. The p-value was calculated through unpaired t-test correction. **G**, **H** Western blot analysis was performed to determine the protein expression levels of HMGA2 in gastric cancer cells HGC27/AGS following Fra-1 overexpression or knockdown. **I** Representative immunohistochemical images showed the expression of HMGA2 in gastric cancer tissues and corresponding paracancerous tissues. 20×: 50 μm; 40×: 20 μm. **J** Immunohistochemical semi-quantitative statistical chart. Normal: 21 cases; Cancer: 23 cases; The p-value was calculated through unpaired t-test correction. **K**, **L** EdU cell proliferation assay combined with flow cytometry was used to evaluate changes in the proliferation ability of gastric cancer cells HGC27/AGS upon Fra-1 overexpression and HMGA2 knockdown. Multiple comparisons were analyzed using ANOVA, and two groups were compared by Wilcoxon rank sum test. **M**, **N** A Transwell cell invasion assay was employed to investigate the effects of Fra-1 overexpression and concurrent HMGA2 knockdown on the invasion capacity of gastric cancer cells HGC27/AGS. Scale bar, 100 μm. Multiple comparisons were analyzed using ANOVA, and two groups were compared by Wilcoxon rank sum test. **O** A scratch healing assay was conducted to assess the impact of Fra-1 overexpression and simultaneous HMGA2 knockdown on the migration ability of the gastric cancer cell line HGC27. Multiple comparisons were analyzed using ANOVA, and two groups were compared by Wilcoxon rank sum test. Scale bar, 200 μm. All experiments were independently repeated three or more times, and the data presented are from representative individual experiments. “ns” indicates no significant difference; “*” indicates *p* < 0.05; “**“ indicates *p* < 0.01; “***” indicates *p* < 0.001; “****“ indicates *p* < 0.0001.

To delve into the potential mechanisms through which Fra-1 contributes to the malignancy of gastric cancer, we conducted a comprehensive proteomic analysis of cells with Fra-1 overexpression, building on our previous research [37]. GO enrichment analysis indicated that the genes with differential expression were significantly associated with tumor cell malignancy (Fig. 2A). We then zeroed in on the top five most upregulated molecules (Fig. 2B) and employed RT-qPCR to validate the mRNA levels of these candidates (ITSN2, IQCB1, MED18, CYP26B1, HMGA2). Our findings revealed that Fra-1 overexpression in gastric cancer cells HGC27/AGS significantly increased the mRNA level of HMGA2 (p < 0.01) (Fig. 2C, D), while Fra-1 knockdown had the opposite effect (p < 0.01) (Fig. 2E, F). Subsequently, Western blot experiments were conducted to assess the impact of Fra-1 on HMGA2 protein expression. The results confirmed that Fra-1 overexpression elevated HMGA2 protein levels, and knockdown reduced it (Fig. 2G), with consistent results observed in the AGS cell line (Fig. 2H). These findings establish that Fra-1 positively regulates HMGA2 expression. Meanwhile, we further examined the expression of HMGA2 in gastric cancer tissues using immunohistochemistry in clinical samples. The results showed that HMGA2 was significantly overexpressed in gastric cancer tissues compared to paracancerous tissues. This finding further suggests that HMGA2 is involved in the malignant progression of gastric cancer (Fig. 2I, J).

To further investigate whether Fra-1 influences the malignant progression of gastric cancer by regulating HMGA2 expression, we overexpressed and knocked down Fra-1 in the gastric cancer cell line HGC27, as well as overexpressed and knocked down Fra-1 while simultaneously knocking down HMGA2. We utilized an EdU cell proliferation assay combined with flow cytometry to detect changes in the proliferation ability of gastric cancer cells. The results indicated that the proliferation rate in the Fra-1 overexpression group was significantly higher than that in the control group (NC group) (p < 0.05). Furthermore, the proliferation rate of gastric cancer cells was significantly reduced after HMGA2 knockdown in the Fra-1 overexpression group (*p* < 0.01) (Fig. 2K). Similar results were observed in the gastric cancer cell line AGS (*p* < 0.01) (Fig. 2M). Upon Fra-1 knockdown, the proliferation rate of the gastric cancer cell line HGC27 decreased compared to the shNC group (*p* < 0.001). Knocking down both Fra-1 and HMGA2 significantly reduced the proliferation rate of the gastric cancer cell line HGC27 compared to the Fra-1 knockdown group alone (*p* < 0.05) (Supplementary Fig. 1E). Consistent results were obtained in the gastric cancer cell line AGS (*p* < 0.05) (Supplementary Fig. 1F). Collectively, these results suggest that overexpression of Fra-1 significantly enhances the proliferation ability of gastric cancer cells, while the co-overexpression of Fra-1 and knockdown of HMGA2 decreases this ability. Knocking down Fra-1 significantly weakens the proliferation ability of gastric cancer cells, and the simultaneous knockdown of both Fra-1 and HMGA2 further reduces this ability, indicating that Fra-1 promotes the proliferation of gastric cancer cells by positively regulating HMGA2.

To further investigate whether Fra-1 affects the invasion and migration abilities of gastric cancer cells by regulating HMGA2 expression, we performed experiments where we overexpressed and knocked down Fra-1 in gastric cancer cells. Additionally, we overexpressed Fra-1 while simultaneously knocking down HMGA2. We utilized the Transwell cell invasion assay and scratch healing assay to assess the invasion and migration capabilities of the HGC27 gastric cancer cell line. The results indicated that the number of invasive cells in the Fra-1 overexpression group was significantly higher than that in the control group (NC group) (*p* < 0.0001), and the scratch width decreased significantly over time (*p* < 0.05). When Fra-1 was overexpressed and HMGA2 was knocked down, the number of invasive cells in the gastric cancer cells was significantly reduced compared to the Fra-1 overexpression group (*p* < 0.0001), and the scratch width increased over time compared to the Fra-1 overexpression group (*p* < 0.05) (Fig. 2L–O). Concurrently, knocking down Fra-1 reduced the number of invasive cells in gastric cancer cells and increased the scratch width over time compared to the shNC group. Knocking down both Fra-1 and HMGA2 significantly reduced the number of invasive cells and increased the scratch width compared to the shFra-1 group (*p* < 0.01) (Fig. 2N, Supplementary Fig. 1J). Furthermore, we repeated the above experiments in the gastric cancer cell line AGS, and the results were consistent (*p* < 0.01) (Supplementary Fig. 1G–I, K). In summary, our findings confirm that Fra-1 promotes the progression of gastric cancer by regulating the expression of HMGA2.

### The binding of Fra-1 and HMGA2 gene promoters regulates their expression and induces M2 polarization in macrophages

**Fig. 3:**
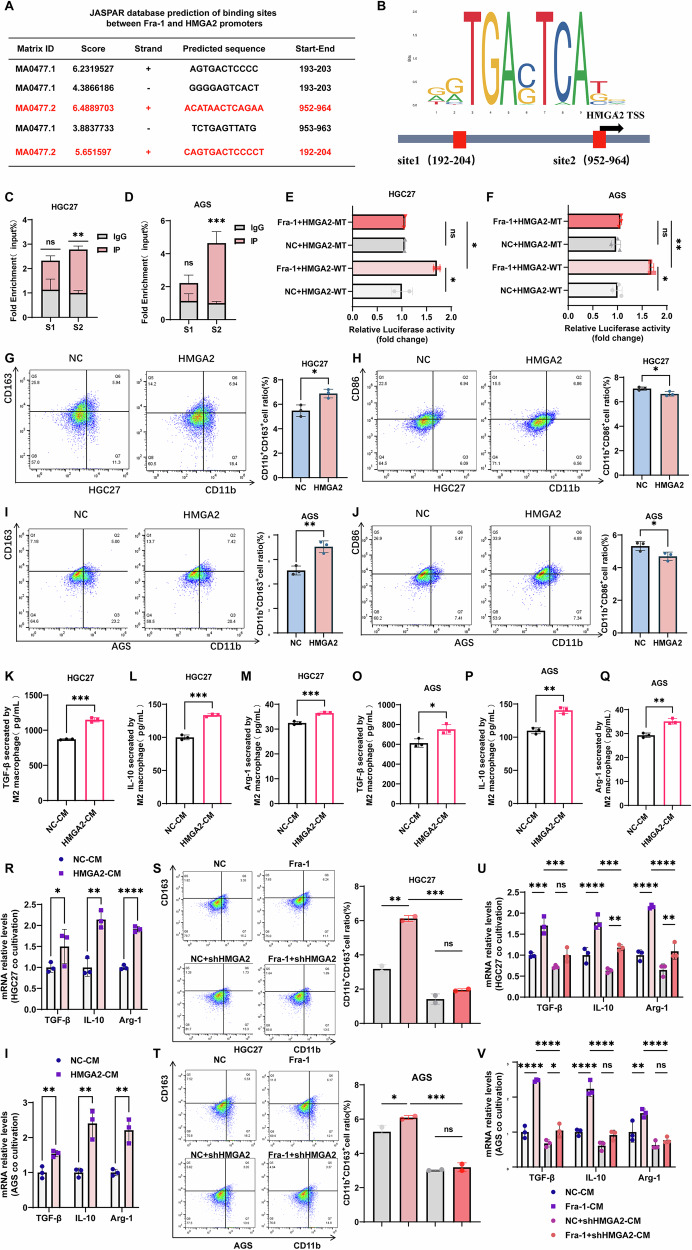
Fra-1 and HMGA2 promoter interaction regulates HMGA2 expression and induces M2 polarization in macrophages. **A**, **B** Prediction of potential binding sites between Fra-1 and the HMGA2 promoter regions using the JASPAR database. **C**, **D** Chromatin Immunoprecipitation followed by quantitative PCR (ChIP-qPCR) assay was conducted to detect the binding of Fra-1 to HMGA2 promoter sites in gastric cancer cells. “IP” indicates enrichment with a Fra-1 antibody; “IgG” indicates enrichment with an IgG antibody. The p-value was calculated through unpaired t-test correction. **E**, **F** Gastric cancer cells were transfected with HMGA2 wild-type (HMGA2-WT) or mutant (HMGA2-MT) dual luciferase reporter gene plasmids after Fra-1 overexpression. Luciferase activity was measured using a dual luciferase reporter gene detection kit after a 48-h incubation. Multiple comparisons were analyzed using ANOVA, and two groups were compared by Wilcoxon rank sum test. **G**–**J** The supernatant from gastric cancer cells overexpressing HMGA2 was used for co-culture with M0 macrophages. M0 macrophages were labeled with CD11b, M1 macrophages with CD86, and M2 macrophages with CD163. Flow cytometry was employed to determine the polarization ratio of M1 and M2 macrophages. The p-value was calculated through unpaired t-test correction. **K**–**Q** The effect of HMGA2 expression in gastric cancer cells on the secretion of M2 macrophage-related cytokines TGF-β, IL-10, and Arg-1 was assessed using an ELISA detection kit following co-culture with M0 macrophages. The p-value was calculated through unpaired t-test correction. **R**, **I** The supernatant from gastric cancer cells overexpressing HMGA2 was used for co-culture with M0 macrophages. RT-qPCR was used to assess the impact of HMGA2 expression on the mRNA levels of M2 macrophage-related cytokines TGF-β, IL-10, and Arg-1 in gastric cancer cells. The p-value was calculated through unpaired t-test correction. **S**, **T** Gastric cancer cell culture supernatants overexpressing Fra-1, and those overexpressing Fra-1 with simultaneous HMGA2 knockdown, were used for co-culture with M0 macrophages labeled with CD11b and M2 macrophages labeled with CD163. Flow cytometry was used to determine the polarization ratio of M2 macrophages. Multiple comparisons were analyzed using ANOVA, and two groups were compared by Wilcoxon rank sum test. **U**, **V** M0 macrophages were co-cultured with the supernatant of gastric cancer cells overexpressing Fra-1 and those overexpressing Fra-1 while knocking down HMGA2. RT-qPCR experiments were conducted to evaluate the effect of Fra-1-regulated HMGA2 expression on the mRNA levels of M2 macrophage-related cytokines TGF-β, IL-10, and Arg-1 in gastric cancer cells. Multiple comparisons were analyzed using ANOVA, and two groups were compared by the Wilcoxon rank sum test. All experiments were independently repeated three or more times, and the data presented are from representative individual experiments. “ns” indicates no significant difference; “*” indicates *p* < 0.05; “**” indicates *p* < 0.01; “***” indicates *p* < 0.001; “****” indicates *p* < 0.0001.

The aforementioned results confirm that Fra-1 promotes the malignant progression of gastric cancer by positively regulating the expression of HMGA2. It is hypothesized that Fra-1 may regulate HMGA2 expression either by interacting with the HMGA2 protein or by binding to its promoter region. To test this hypothesis, we initially conducted Co-Immunoprecipitation (Co-IP) experiments to determine if there was a protein interaction between Fra-1 and HMGA2. The findings indicated no interaction between the Fra-1 and HMGA2 proteins (Supplementary Fig. 1C, D). Subsequently, we utilized the JASPAR database to predict the presence of two potential binding sites within the promoter regions of the Fra-1 and HMGA2 genes (Fig. 3A, B, Supplementary Table 4). We then performed Chromatin Immunoprecipitation quantitative Polymerase Chain Reaction (ChIP-qPCR) experiments to verify the binding of Fra-1 to the predicted sites of the HMGA2 promoter. The results revealed that the second predicted site of HMGA2 was significantly enriched in gastric cancer cells (p < 0.01) (Fig. 3C, D). To further confirm the binding of Fra-1 to the second site of the HMGA2 gene promoter region, we constructed both wild-type (PGL3-Basic-HMGA2-promoter-WT) and mutant (PGL3-Basic-HMGA2-promoter-MT) dual luciferase reporter gene vectors for the HMGA2 gene and transfected them into gastric cancer cells. A dual luciferase reporter gene detection kit was employed to measure their activity. The results demonstrated that in gastric cancer cells overexpressing Fra-1, the luciferase activity of the HMGA2-WT group was significantly increased, whereas the mutated HMGA2-MT group exhibited a significant decrease (Fig. 3E, F). Meanwhile, to further verify the specificity of Fra-1 binding to the HMGA2 promoter II site, we constructed a biotin-labeled probe targeting the HMGA2 promoter II site. The direct and specific binding of Fra-1 to this site was confirmed through an EMSA (electrophoretic mobility shift assay) experimental assay (Supplementary Fig. 2A, B). These findings further confirm that Fra-1 binds to the second site of the HMGA2 promoter and regulates its expression through transcriptional activation (p < 0.05).

Previous research has confirmed that high mobility group A2 (HMGA2), as an oncogene, plays a significant role in macrophage recruitment and polarization [38]. To verify whether HMGA2 expression in gastric cancer cells influences macrophage polarization, we initially overexpressed HMGA2 in the gastric cancer cell line HGC27 and prepared conditioned medium (CM). Through in vitro co-culture experiments, we utilized flow cytometry to analyze the polarization ratio of M1 and M2 macrophages. The results indicated that the proportion of M2 polarization induced by the supernatant of HMGA2-overexpressing gastric cancer cells was significantly higher than that of the control group (NC group) (p < 0.05) (Fig. 3G). Conversely, the proportion of M1 polarization induced by the supernatant of HMGA2-overexpressing gastric cancer cells was significantly lower than that of the control group (NC group) (*p* < 0.05) (Fig. 3H). Consistent results were observed in the gastric cancer cell line AGS (*p* < 0.05) (Fig. 3I, J).

Additionally, we employed an ELISA detection kit and RT-qPCR technology to assess the impact of conditioned medium from HMGA2-highly-expressing gastric cancer cells on the secretion of M2 macrophage-related cytokines, including TGF-β, IL-10, and Arg-1. The findings revealed that overexpression of HMGA2 in gastric cancer cell culture supernatant led to increased secretion of M2 macrophage marker cytokines TGF-β, IL-10, and Arg-1 (*p* < 0.001), as well as elevated mRNA levels (*p* < 0.05) (Fig. 3K–M, R). Similar results were obtained in the gastric cancer cell line AGS (p < 0.05) (Fig. 3O–Q, I). In summary, the results suggest that gastric cancer cells with high HMGA2 expression promote M2 polarization of macrophages.

To further investigate whether Fra-1 influences the polarization of macrophages within the tumor microenvironment by regulating HMGA2 expression in gastric cancer cells, we simultaneously overexpressed Fra-1 and knocked down HMGA2 in the gastric cancer cell line HGC27. Utilizing in vitro co-culture experiments, we employed flow cytometry to assess the polarization ratio of M1 and M2 macrophages. Our findings revealed that the supernatant from HGC27 cells overexpressing Fra-1 induced a higher level of M2 polarization in macrophages compared to the control group (NC group) (p < 0.01). However, when HMGA2 was knocked down in cells overexpressing Fra-1, the M2 polarization was significantly reduced compared to the Fra-1 overexpression group alone (p < 0.001) (Fig. 3S). Conversely, the supernatant from HGC27 cells overexpressing Fra-1 induced a lower proportion of M1 polarization compared to the control group (*p* < 0.01), and knocking down HMGA2 in these cells resulted in a significantly higher proportion of M1 polarization compared to the Fra-1 overexpression group (*p* < 0.001) (Supplementary Fig. 3A). Consistent results were observed in the gastric cancer cell line AGS (Fig. 3T, Supplementary Fig. 3B).

Additionally, we further elucidated the impact of Fra-1 on the secretion of M2 macrophage-related cytokines, including TGF-β, IL-10, and Arg-1, in the gastric cancer cell line HGC27 by regulating HMGA2 expression. The overexpression of Fra-1 in gastric cancer cell culture supernatant led to increased mRNA levels and secretion of M2 macrophage marker cytokines TGF-β, IL-10, and Arg-1. In contrast, the overexpression of Fra-1 combined with the knockdown of HMGA2 resulted in decreased mRNA levels and secretion of these M2 macrophage marker cytokines (*p* < 0.05) (Fig. 3U, Supplementary Fig. 3C–E). Similar results were obtained in the gastric cancer cell line AGS (*p* < 0.05) (Fig. 3V, Supplementary Fig. 3F–H). In conclusion, our results demonstrate that gastric cancer cells with high HMGA2 expression promote M2 polarization of macrophages. Overall, our findings confirm that Fra-1 facilitates M2 polarization of macrophages in gastric cancer cells by regulating the expression of HMGA2.

### Fra-1 promotes the binding of CCL2 and CCR2 by regulating HMGA2 expression, thereby inducing M2 polarization in macrophages

**Fig. 4:**
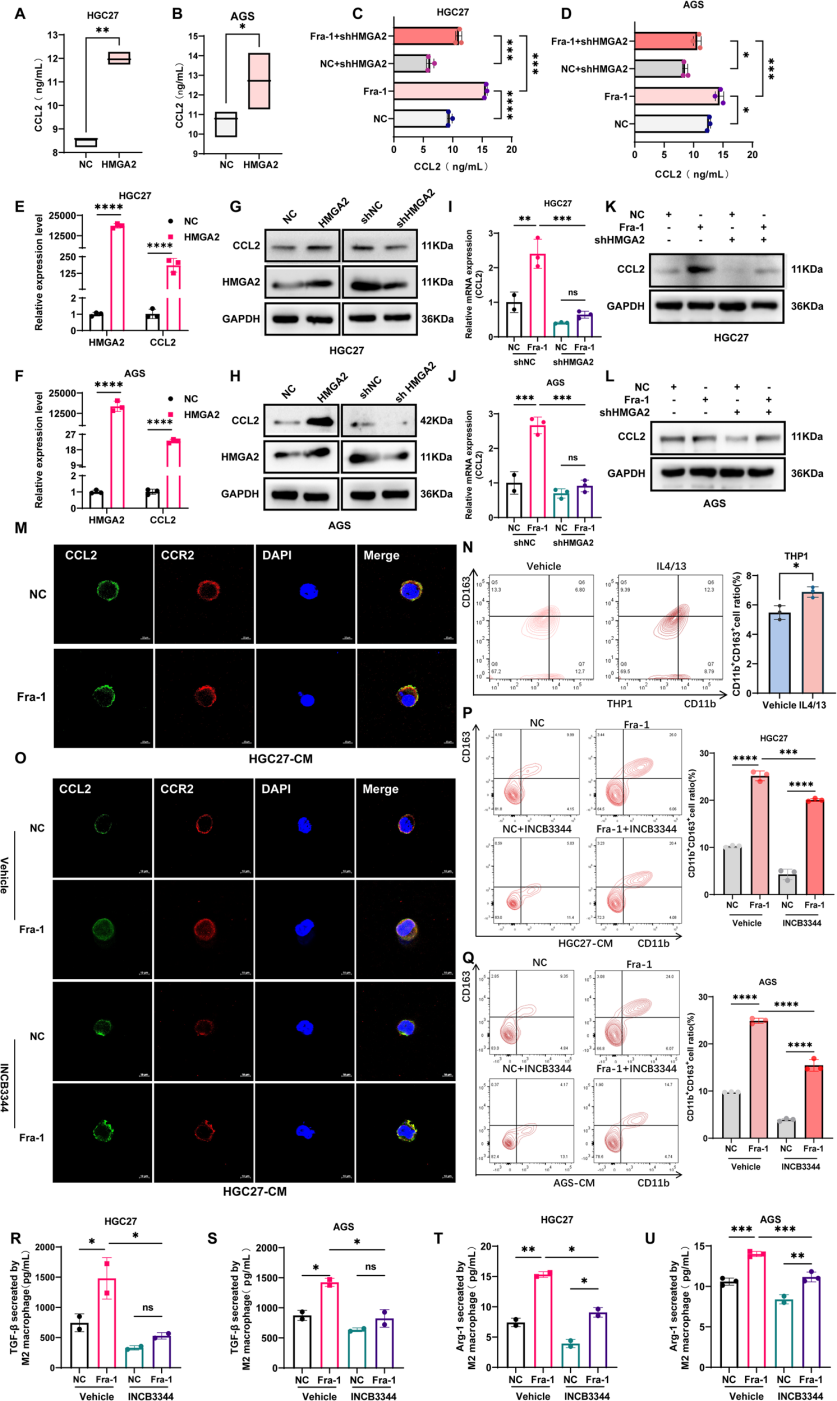
Fra-1 regulation of HMGA2 expression promotes CCL2 binding to CCR2 and induces macrophage M2-type polarization. **A**, **B** HMGA2 was overexpressed in gastric cancer cells, and the effect of HMGA2 on CCL2 secretion was detected using an ELISA assay kit. The p-value was calculated through unpaired t-test correction. **C**, **D** In gastric cancer cells, Fra-1 was overexpressed, and Fra-1 was also overexpressed while HMGA2 was knocked down. The effect of Fra-1-regulated HMGA2 expression on CCL2 secretion was detected using an ELISA assay kit. Multiple comparisons were analyzed using ANOVA, and two groups were compared by Wilcoxon rank sum test. **E**, **F** In gastric cancer cells HGC27, HMGA2 overexpression was performed, and the effect of HMGA2 on CCL2 mRNA levels was detected using RT-qPCR. The p-value was calculated through unpaired t-test correction. **G**, **H** In gastric cancer cells HGC27, HMGA2 overexpression was performed, and the effect of HMGA2 on CCL2 protein expression levels was detected using Western blot. **I**, **J** In gastric cancer cells AGS, HMGA2 overexpression was performed, and the effect of HMGA2 on CCL2 mRNA levels was detected using RT-qPCR. Multiple comparisons were analyzed using ANOVA, and two groups were compared by Wilcoxon rank sum test. **K**, **L** In gastric cancer cells AGS, HMGA2 overexpression was performed, and the effect of HMGA2 on CCL2 protein expression levels was detected using Western blot. **M** M0 macrophages were co-cultured with conditioned medium from gastric cancer cells overexpressing Fra-1, and the co-localization of CCL2 secreted by gastric cancer cells and CCR2 expressed by macrophages was detected using laser confocal microscopy. **N** M0 macrophages were induced to polarize to the M2 type by adding IL4 (20 ng/mL) and IL13 (20 ng/mL) to the medium, and the induction efficiency was detected using flow cytometry. The p-value was calculated through unpaired t-test correction. **O** Successfully induced M2-type macrophages were co-cultured with conditioned medium from gastric cancer cells overexpressing Fra-1, with (or without) the addition of the CCR2 antagonist INCB3344 (5 nM), and the co-localization of CCL2 secreted by gastric cancer cells and CCR2 expressed by macrophages was detected using laser confocal microscopy. **P**, **Q** Successfully induced M2-type macrophages were co-cultured with conditioned medium from gastric cancer cells overexpressing Fra-1, with (or without) the CCR2 antagonist INCB3344, and the percentage of M2-type macrophages polarized was detected using flow cytometry. Multiple comparisons were analyzed using ANOVA, and two groups were compared by Wilcoxon rank sum test. **R**–**U** Successfully induced M2-type macrophages were co-cultured with conditioned medium from gastric cancer cells overexpressing Fra-1, with (or without) the addition of the CCR2 antagonist INCB3344, and the secretion of the M2-type macrophage marker cytokines TGF-β, Arg-1, and IL-10 was detected using an ELISA assay kit. Multiple comparisons were analyzed using ANOVA, and two groups were compared by Wilcoxon rank sum test. All experiments were performed with three or more independent replications, and the data shown are from representative individual experiments. “ns” indicates not significantly different; “*“ indicates *p* < 0.05; “**“ indicates *p* < 0.01; “***“ indicates *p* < 0.001; “****“ indicates *p* < 0.0001.

Previous research indicates that HMGA2 can enhance macrophage recruitment and M2 polarization in the tumor microenvironment by influencing the secretion of CCL2 in tumor cells [38]. To ascertain whether HMGA2 facilitates macrophage recruitment and M2 polarization in gastric cancer cells through the induction of CCL2 secretion, we initially overexpressed HMGA2 in the gastric cancer cell line HGC27. We then employed an ELISA detection kit and RT-qPCR experiments to measure the secretion of CCL2 in these cells. Our findings demonstrated that the overexpression of HMGA2 led to a significant increase in both CCL2 secretion and mRNA levels in gastric cancer cells (p < 0.01) (Fig. 4A–E). This was corroborated by results from the gastric cancer cell line AGS, which also showed a significant increase (*p* < 0.05) (Fig. 4B–F). Concurrently, Western blot experiments were conducted to further evaluate the impact of HMGA2 on the expression levels of CCL2 protein in the gastric cancer cell line HGC27. The results revealed that overexpression of HMGA2 significantly upregulated CCL2 protein levels, and these levels were significantly reduced after HMGA2 knockdown (Fig. 4G). Consistent results were observed in the AGS gastric cancer cells (Fig. 4H). In summary, the aforementioned results suggest that the overexpression of HMGA2 promotes CCL2 secretion in gastric cancer cells, potentially contributing to macrophage recruitment and M2 polarization.

To further ascertain whether Fra-1 in gastric cancer cells influences the secretion of CCL2 by regulating HMGA2 expression, we conducted experiments in which we overexpressed Fra-1 and simultaneously knocked down HMGA2 in the gastric cancer cell line HGC27. We then used an ELISA assay kit and RT-qPCR to measure the secretion of CCL2 in these cells. Our findings indicated that the overexpression of Fra-1 led to a significant increase in both CCL2 secretion and mRNA levels in gastric cancer cells. In contrast, when Fra-1 was overexpressed while HMGA2 was knocked down, there was a significant decrease in CCL2 secretion and mRNA levels compared to the group overexpressing Fra-1 alone (p < 0.01) (Fig. 4C, I). Similar results were observed in the gastric cancer cell line AGS (p < 0.05) (Fig. 4D, J). Additionally, we utilized Western blot assays to further investigate the impact of Fra-1 on the protein expression levels of CCL2 by regulating HMGA2 in gastric cancer cells HGC27. The results demonstrated that the protein levels of CCL2 were significantly upregulated by the overexpression of Fra-1 and were significantly downregulated when Fra-1 was overexpressed while HMGA2 was knocked down (Fig. 4K). Consistent results were obtained in the AGS gastric cancer cells (Fig. 4L). In summary, these results confirm that Fra-1 promotes CCL2 secretion in gastric cancer cells by regulating the expression of HMGA2.

To further elucidate whether Fra-1 induces macrophage polarization by affecting CCL2 secretion from gastric cancer cells, we conducted a series of experiments. Initially, we detected the co-localization of CCL2 secreted by gastric cancer cells with CCR2 expressed by macrophages using laser confocal microscopy. The co-localization of CCL2 and CCR2 was significantly enhanced after co-culturing macrophages with the supernatant from Fra-1-overexpressing gastric cancer cells (Fig. 4M, Supplementary Fig. 3I). Next, we induced M2 polarization in M0 macrophages by adding IL4 and IL13 to their medium and confirmed the induction’s success using flow cytometry (Fig. 4N). We then co-cultured these M2-type macrophages with the conditioned medium from Fra-1-overexpressing gastric cancer cells, with or without the CCR2 antagonist INCB3344. The co-localization of CCL2 with CCR2 was significantly enhanced in the presence of Fra-1-overexpressing cancer cell supernatants and was significantly reduced by the addition of INCB3344 (Fig. 4O). Consistent results were observed with AGS cells (Supplementary Fig. 3J). Furthermore, we found that the conditioned medium from Fra-1-overexpressing gastric cancer cells co-cultured with M2-type macrophages significantly increased the percentage of M2 macrophage polarization, as detected by flow cytometry (p < 0.0001) (Fig. 4P). This effect was diminished by the addition of INCB3344, and similar results were obtained with AGS cells (p < 0.001) (Fig. 4Q).

Additionally, we co-cultured M2-type macrophages with the conditioned medium from Fra-1-overexpressing gastric cancer cells, with or without the CCR2 antagonist INCB3344, and measured the secretion of M2 macrophage marker cytokines TGF-β, Arg-1, and IL-10 using an ELISA assay kit. The conditioned medium from Fra-1-overexpressing cancer cells increased the secretion of these cytokines, and this increase was significantly reduced by the addition of INCB3344 (p < 0.05) (Fig. 4R–U, Supplementary Fig. 3K, L). AGS cells showed consistent results. In summary, our results suggest that Fra-1 induces macrophage polarization towards the M2 type by promoting CCL2 secretion from gastric cancer cells, which then binds to the macrophage receptor CCR2. Collectively, our experimental data confirm that Fra-1 in gastric cancer cells enhances CCL2 secretion by regulating HMGA2 expression, thereby binding to macrophage receptor CCR2 and inducing M2 polarization.

### M2-type macrophages promote gastric cancer progression by inducing angiogenesis through secretion of VEGF

**Fig. 5:**
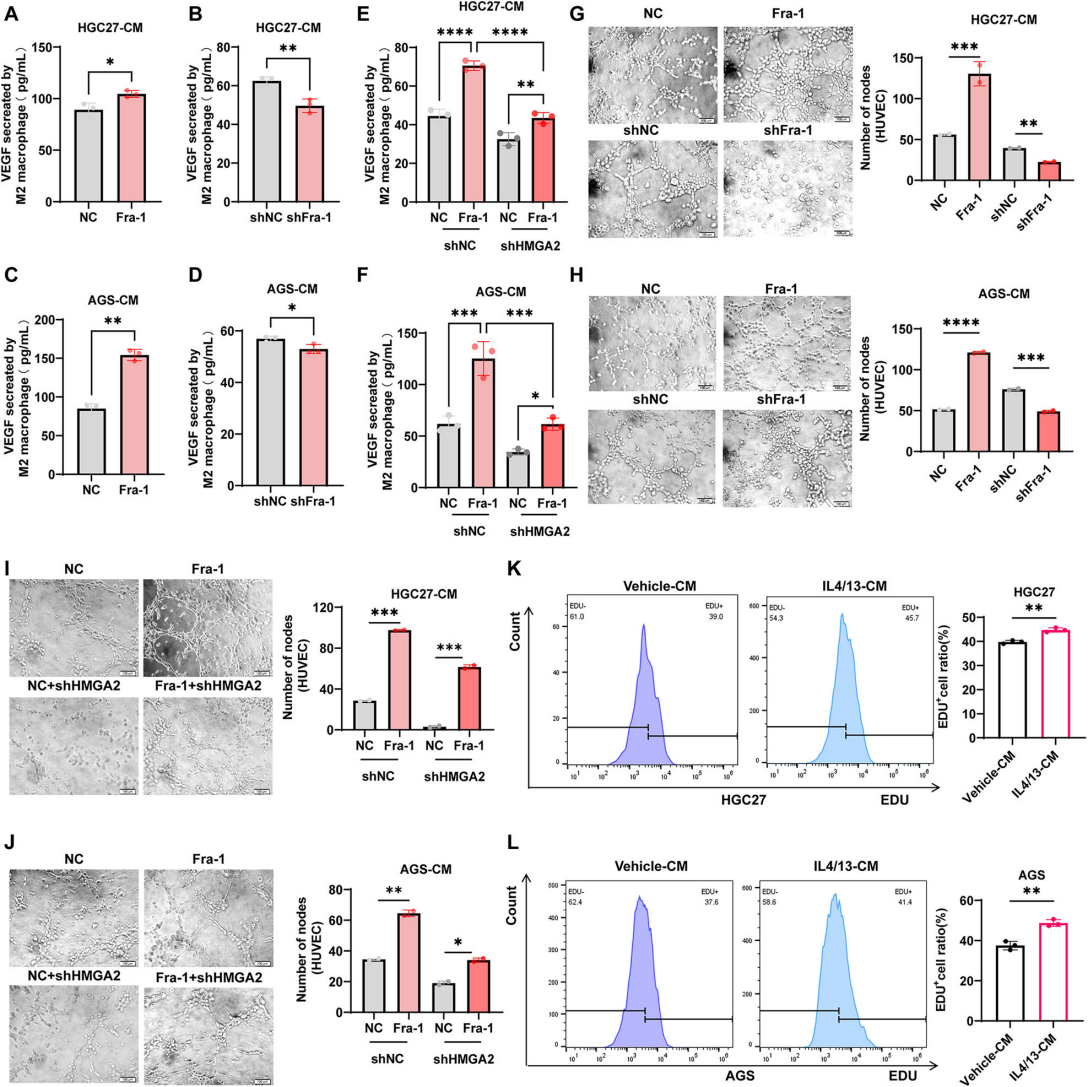
M2-type macrophages promote gastric cancer progression by secreting VEGF to induce angiogenesis. **A**–**D** Fra-1 was overexpressed or knocked down in gastric cancer cells HGC27/AGS. After 48 h, the cell culture supernatant was collected to prepare conditioned medium. Following the induction of M0 macrophages to M2 polarization using IL4 and IL13, the conditioned medium was co-cultured with the M2 macrophages for an additional 48 h. The ELISA detection kit was used to measure the secretion of vascular endothelial growth factor (VEGF) by M2-type macrophages. The p-value was calculated through unpaired t-test correction. **E**, **F** Fra-1 was overexpressed in gastric cancer cells HGC27/AGS, with or without HMGA2 knockdown. After 48 h, the cell culture supernatant was collected to prepare conditioned medium. The M0 macrophages were induced to polarize towards M2 using IL4 and IL13, and then co-cultured with the conditioned medium for 48 h. The secretion of VEGF from M2 macrophages was detected using an ELISA kit. Multiple comparisons were analyzed using ANOVA, and two groups were compared by Wilcoxon rank sum test. **G**, **H** Fra-1 was overexpressed or knocked down in gastric cancer cells HGC27/AGS. The cell culture supernatant was collected after 48 h to prepare conditioned medium. After inducing M0 macrophages to polarize to M2 using IL4 and IL13, the conditioned medium was co-cultured with M2 macrophages. The cell culture supernatant was collected after an additional 48 h to prepare macrophage-conditioned medium. This was further co-cultured with vascular endothelial cells for 48 h, and the tubule formation ability of the vascular endothelial cells was assessed using a tubule formation assay. The p-value was calculated through unpaired t-test correction. **I**, **J** Fra-1 was overexpressed in gastric cancer cells HGC27/AGS, with or without concomitant HMGA2 knockdown. Macrophage-conditioned medium was prepared and further co-cultured with vascular endothelial cells. After 48 h, the tubule formation ability of the vascular endothelial cells was detected using a tubule formation assay. The p-value was calculated through unpaired t-test correction. **K**, **L** M0 macrophages were induced or not induced to polarize towards M2 using IL4 and IL13. After 48 h, the cell culture supernatants were collected, and macrophage-conditioned medium was prepared. This was further co-cultured with gastric cancer cells, and the effect of M2 macrophages on the proliferative ability of gastric cancer cells was detected using EdU incorporation combined with flow cytometry. The p-value was calculated through unpaired t-test correction. All experiments were performed with three or more independent replications, and the data shown are from representative individual experiments. “ns” indicates no significant difference; “*” indicates *p* < 0.05; “**“ indicates *p* < 0.01; “***” indicates *p* < 0.001; “****” indicates *p* < 0.0001.

M2-type macrophages are known to influence the progression of malignant tumors through pro-angiogenesis, lymphangiogenesis, and suppression of adaptive immunity [39]. To explore whether M2-type macrophages facilitate the malignant progression of gastric cancer by inducing angiogenesis, we conducted a series of experiments. Initially, we used PMA to induce the transformation of THP1 into M0 macrophages. Subsequently, we added IL-4/IL-13 to induce the transformation of M0 macrophages into M2 macrophages and performed RNA-seq analysis. This revealed the presence of 11 differentially upregulated angiogenesis-associated factors in M2 macrophages, among which we focused on the top three angiogenic factors: MMP12, MMP7, and VEGF (Supplementary Fig. 4A). To further verify the effect of Fra-1 expression in gastric cancer cells on the expression of these three angiogenesis-related factors in M2 macrophages in vitro, we first overexpressed Fra-1 in gastric cancer cells and collected the conditioned medium. We then co-cultured this conditioned medium with M2 macrophages and detected the expression levels using RT-qPCR experiments. The results showed that the VEGF mRNA expression level was significantly elevated compared to the negative control (NC) group (Supplementary Fig. 4B, C). Next, we overexpressed Fra-1 in gastric cancer cells and simultaneously knocked down HMGA2 to investigate whether Fra-1 affects the expression of angiogenesis-related factors in M2 macrophages through HMGA2. RT-qPCR results demonstrated that the VEGF mRNA expression level in M2 macrophages co-cultured with conditioned medium from Fra-1-overexpressing gastric cancer cells was significantly elevated. However, when HMGA2 was knocked down in these cells, the VEGF mRNA expression level in M2 macrophages was reduced (Supplementary Fig. 4D, E). These findings suggest that Fra-1 in gastric cancer cells affects the secretion of VEGF from M2 macrophages in the microenvironment through the modulation of HMGA2, thereby influencing angiogenesis. To further validate the above results, we overexpressed and knocked down Fra-1 in the gastric cancer cell line HGC27. After 48 h, we collected the cell culture supernatants to prepare conditioned medium, which was then co-cultured with M2-type macrophages. Following another 48 h, we utilized an ELISA assay kit to measure the secretion of vascular endothelial growth factor (VEGF) by M2 macrophages. Our findings revealed that the conditioned medium from Fra-1-overexpressing gastric cancer cells led to an increase in VEGF secretion from M2 macrophages, whereas the Fra-1 knockdown group exhibited a decrease in VEGF secretion (Fig. 5A, B). Consistent results were observed in the gastric cancer cell line AGS (Fig. 5C, D).

Subsequently, we overexpressed Fra-1 and simultaneously knocked down HMGA2 in the gastric cancer cell line HGC27, prepared conditioned medium, and performed in vitro co-culture experiments with M2-type macrophages. The results indicated that the culture supernatants from Fra-1-overexpressing gastric cancer cells increased VEGF secretion from M2-type macrophages. In contrast, the culture supernatants from cells overexpressing Fra-1 while also knocking down HMGA2 significantly reduced VEGF secretion compared to the Fra-1 overexpression group alone (Fig. 5E). Similar results were obtained with the AGS gastric cancer cells (Fig. 5F). These results suggest that Fra-1 enhances VEGF secretion from M2-type macrophages by regulating HMGA2 expression in gastric cancer cells, thereby potentially promoting angiogenesis and the malignant progression of gastric cancer.

To further investigate whether Fra-1 induces angiogenesis by promoting M2 polarization of macrophages, we conducted a series of experiments. Initially, we overexpressed and knocked down Fra-1 in gastric cancer cells HGC27 and AGS to prepare conditioned medium. This conditioned medium was then co-cultured with M2-type macrophages to prepare macrophage-conditioned medium, which was further co-cultured with human umbilical vein endothelial cells (HUVECs). After 48 h, we collected the cells and used a tubule formation assay to assess the tubule formation ability of the vascular endothelial cells. The results indicated that the tubule formation ability of vascular endothelial cells was enhanced by the conditioned medium from M2 macrophages overexpressing Fra-1, while it was attenuated after Fra-1 knockdown (P < 0.01) (Fig. 5G, H). Subsequently, we overexpressed Fra-1 and simultaneously knocked down HMGA2 in gastric cancer cells HGC27 and AGS. We prepared conditioned medium with macrophages and further co-cultured it with HUVECs. After 48 h, we again used a tubule formation assay to detect the ability of vascular endothelial cells to form tubules. The conditioned medium from gastric cancer cells overexpressing Fra-1, when co-cultured with M2 macrophages, resulted in enhanced vascular endothelial cell tubule formation. However, this effect was diminished when Fra-1 was overexpressed concurrently with HMGA2 knockdown (P < 0.05) (Fig. 5I, J). These results suggest that Fra-1 in gastric cancer cells enhances the ability of M2 macrophages to induce vascular endothelial cell tubule formation by regulating HMGA2. Furthermore, we co-cultured M2 macrophage-conditioned medium with gastric cancer cells and used EdU combined with flow cytometry to detect the effect of M2 macrophages on the proliferative ability of gastric cancer cells. The proportion of proliferating gastric cancer cells was significantly increased after co-culture with M2 macrophages induced by IL4 and IL13, compared to the control group (Fig. 5K, L). Our results suggest that Fra-1 in gastric cancer cells promotes M2-type macrophage polarization-induced angiogenesis by regulating the expression of HMGA2, which in turn promotes the malignant progression of gastric cancer cells.

### In vivo experiments confirm that Fra-1 promotes gastric cancer progression by inducing macrophage polarization

**Fig. 6:**
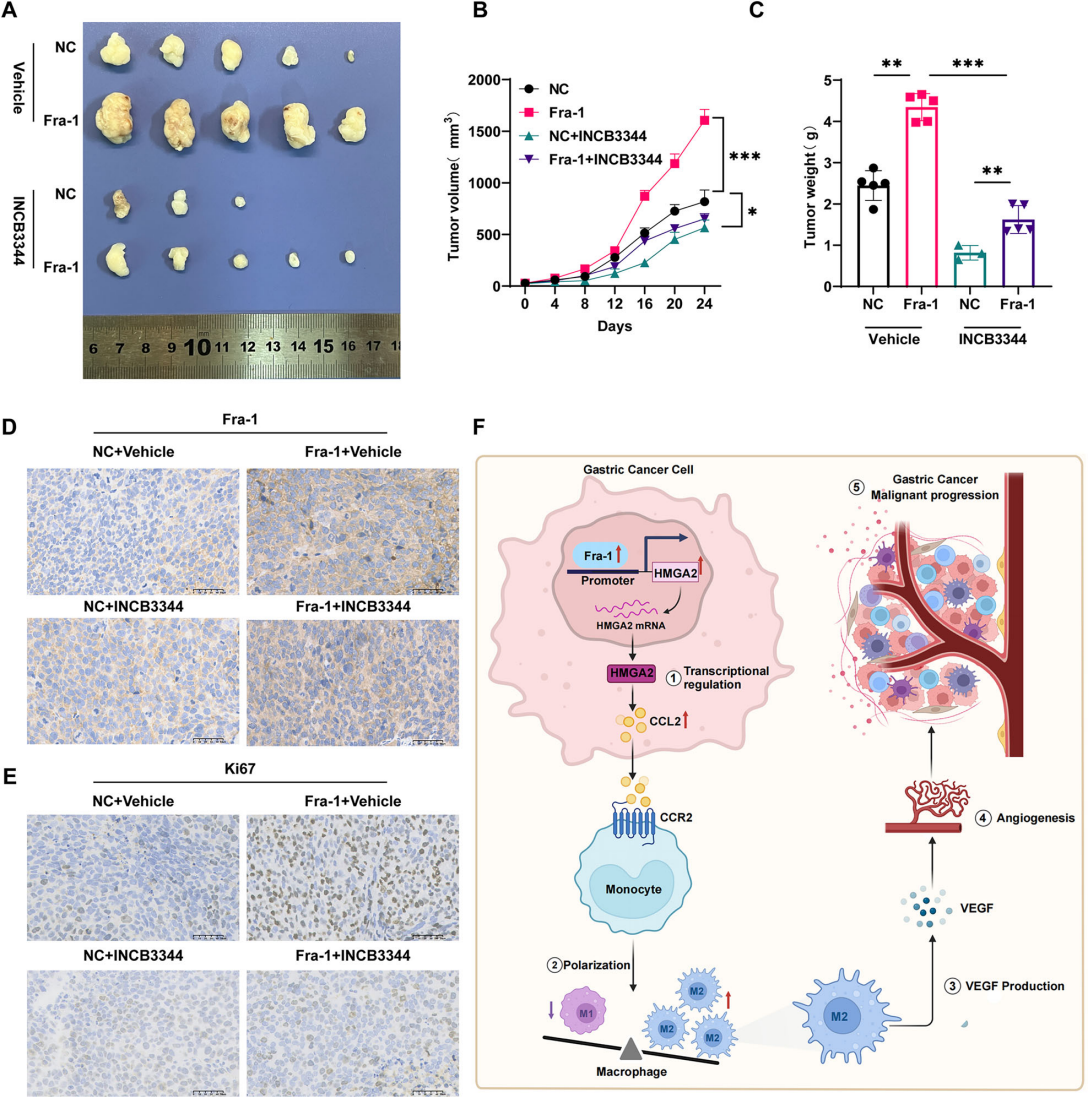
Fra-1 enhances gastric cancer progression by inducing macrophage polarization. **A**–**C** Graphical representation of the morphological characteristics of subcutaneous xenograft tumors, growth rate of tumor volume, and histograms of tumor weight for the following groups: NC (negative control), Fra-1 (Fra-1 overexpression), NC + INCB3344 (treatment with CCR2 antagonist INCB3344), and Fra-1 + INCB3344 (Fra-1 overexpression with INCB3344 treatment). Survival plots were calculated using Log-rank (Mantel-Cox) test for p-value. Multiple comparisons were analyzed using ANOVA, and two groups were compared by Wilcoxon rank sum test. **D** Immunohistochemistry (IHC) analysis to assess the expression efficiency of Fra-1 in the NC, Fra-1, NC + INCB3344, and Fra-1 + INCB3344 groups. Scale bar is indicated. **E** IHC analysis to evaluate the expression efficiency of Ki67, a proliferation marker, in the NC, Fra-1, NC + INCB3344, and Fra-1 + INCB3344 groups. Scale bar is indicated. **F** Schematic diagram illustrating the proposed mechanism by which Fra-1 promotes gastric cancer progression through the induction of macrophage polarization. All experiments were conducted with three or more independent replications. “ns” denotes no significant difference; “*” denotes *p* < 0.05; “**“ denotes *p* < 0.01; “***” denotes *p* < 0.001; “****” denotes *p* < 0.0001.

To further confirm that Fra-1 promotes gastric cancer progression by inducing macrophage polarization, we conducted in vivo xenograft model experiments using BALB/c nude mice. A total of 20 mice were randomly divided into four groups. HGC27 gastric cancer cells transfected with either the Fra-1 plasmid or a negative control were injected into the axillary subcutis of the mice. Once tumors were established, the mice were treated with intraperitoneal injections of the CCR2 antagonist INCB3344 (10 mg/kg) or with no injection. Tumor size was measured every four days, and after 24 days, the mice were sacrificed. The subcutaneous tumor tissues were then removed, photographed, and weighed. The results indicated that the Fra-1 overexpression group had the largest tumor volume, the fastest growth rate, and the heaviest weight compared to the control group. Treatment with INCB3344 led to a reduction in tumor volume, slower growth rate, and lighter weight. Notably, two mice in the negative control (NC) group showed regression of subcutaneous tumors following INCB3344 administration (Fig. 6A–C). Furthermore, immunohistochemical analysis revealed that tumors overexpressing Fra-1 had significantly higher Ki67 indices, and INCB3344 injection significantly reduced the Ki67 index levels (Fig. 6D, E). These findings suggest that Fra-1 overexpression enhances gastric cancer cell proliferation, and this effect is substantially diminished when CCR2 expression in the tumor microenvironment is inhibited. Collectively, the in vivo experiments substantiated that Fra-1 induces macrophage polarization, thereby promoting gastric cancer progression.

## Discussion

Gastric cancer ranks 6th among malignant tumors in terms of incidence and 3rd in terms of mortality, making it one of the leading causes of cancer-related deaths globally [38, 40]. Although significant progress has been made in the early diagnosis, surgical resection, and adjuvant chemotherapy of gastric cancer, the morbidity and mortality rates remain high due to the lack of early diagnostic markers [41, 42]. Therefore, there is an urgent need to better understand the pathogenesis of gastric cancer and to identify new molecular targets for its early diagnosis, treatment, and prevention. The activation of oncogenes and the inactivation of tumor suppressor genes play crucial roles in the development of gastric cancer [43]. Fra-1, a member of the Fos family, is an important nuclear transcription factor that regulates normal cell growth, differentiation, and apoptosis [44, 45]. It is highly expressed in many malignant tumors, including gastric, liver, and thyroid cancers, where it plays a significant role in cell proliferation, invasion, and metastasis [44, 46]. In our previous study, we found that Fra-1 was highly expressed in gastric cancer (GC) tissues compared to adjacent non-neoplastic tissues; overexpression of Fra-1 in AGS gastric cancer cells resulted in a significant decrease in apoptotic cells and an increase in the S-phase fraction; moreover, overexpression of Fra-1 in AGS gastric cancer cells affected the expression levels of PI3K, AKT, MDM2, and p53 in vivo [6]. Fra-1 is closely associated with the progression of gastric cancer, but its detailed mechanism warrants further elucidation.

In this study, we aimed to further elucidate the specific mechanisms by which Fra-1 affects the malignant progression of gastric cancer. Initially, we validated the expression of five molecules that were most significantly up-regulated in the proteomic analysis following the overexpression of Fra-1 in gastric cancer cells [37]. The results indicated that HMGA2, a member of the HMGA family, was significantly up-regulated after the overexpression of Fra-1 in gastric cancer cells. Conversely, HMGA2 was significantly down-regulated in gastric cancer cells following the knockdown of Fra-1. These findings suggest that Fra-1 positively regulates the expression of HMGA2 in gastric cancer cells. Meanwhile, co-immunoprecipitation (Co-IP) results showed that Fra-1 does not interact with the HMGA2 protein. However, chromatin immunoprecipitation quantitative polymerase chain reaction (ChIP-qPCR) and dual luciferase reporter assays confirmed that Fra-1 binds to the HMGA2 promoter. Our results suggest that Fra-1 activates HMGA2 expression through transcriptional regulation, thereby affecting its function.

HMGA2 is a member of the high mobility group A (HMGA) family of proteins. HMGA proteins play crucial roles in biological processes such as gene transcription regulation, chromatin remodeling, DNA repair, and RNA processing [47–50]. HMGA2 has been found to be re-expressed in almost all human malignancies and promotes tumorigenesis and progression through multiple mechanisms [51]. In colorectal cancer, researchers discovered that HMGA2 directly binds to the STAT3 promoter to activate its transcription, subsequently inducing CCL2 secretion, which promotes macrophage recruitment and colorectal cancer progression [38]. It is evident that HMGA2 is closely associated with macrophage polarization in the tumor microenvironment. In this study, we verified whether HMGA2 in gastric cancer cells affects macrophage polarization and found that the culture supernatant of gastric cancer cells overexpressing HMGA2 induced a significant increase in the proportion of M2-type macrophage polarization, as well as an increase in the secretion of M2-type macrophage marker cytokines, such as TGF-β, IL-10, and Arg-1. This suggests that gastric cancer cells with high HMGA2 expression promote a microenvironment with M2-type macrophage polarization. The results indicate that high HMGA2 expression in gastric cancer cells promotes the polarization of M2-type macrophages in the microenvironment, and further demonstrate that Fra-1 in gastric cancer cells induces the polarization of M2-type macrophages in the microenvironment through the regulation of HMGA2 expression and the secretion of CCL2.

Meanwhile, this study found that CCL2, after secretion, can directly bind to CCR2 on the surface of macrophages, thereby inducing their M2 polarization. When the CCL2 antagonist INCB3344 was used, the proportion of CCL2 binding to CCR2 was significantly reduced, and the proportion of M2 polarization decreased. These results further confirmed that CCL2 induces macrophage M2 polarization by binding to CCR2. INCB3344 is a selective CCR2 antagonist, and the current study demonstrated that it has high affinity for CCR2, with an IC50 of 5.1 nM for human-derived CCR2 and 9.5 nM for mouse-derived CCR2. However, as a small molecule drug, its potential off-target effects need to be carefully evaluated during drug development. Despite its high selectivity for CCR2 (with 5000-fold lower affinity for HRAS/NRAS), other studies have suggested that some CCR antagonists may cross-react with related receptors due to structural similarity, such as CCR5, CCR4, and non-chemokine GPCRs. Therefore, future studies need to further utilize high-throughput screening techniques to assess its effects on non-targets and to determine its metabolic enzyme profiles and drug-drug interaction potential. Through multidimensional evaluation, the off-target risk of INCB3344 can be minimized, and its development into a safer therapeutic drug can be promoted.

In addition, research has reported that, in addition to CCL2, multiple chemokines affect macrophage recruitment and polarization. For example, EZH2 (an enhancer of Zeste homolog 2) can enhance the expression of chemokine CCL5, leading to macrophage recruitment (10.1002/bab.1875). Meanwhile, CCL5 plays an important role in mediating macrophage polarization induced by integrin β8 (ITGβ8) (10.1002/advs.202406865). In periodontal inflammation (PI), it has also been found that the production of IL-1β promotes the expression of CCL5, CXCL12, CCL2, and CXCL5, which recruit macrophages and ultimately enable the inflammatory site to form a pre-metastatic niche (10.1038/s41388-019-1084-Z). In summary, in addition to CCL2, chemokines such as CCL5, CXCL12, and CXCL5 are all closely related to macrophage recruitment. Therefore, it remains to be explored in the future whether Fra-1, via HMGA2 in gastric cancer cells, affects the secretion of other macrophage-recruitment-related chemokines in addition to CCL2. Additionally, it is worth investigating whether the transcription factor Fra-1 can directly transcriptionally regulate these chemokines, thereby promoting macrophage recruitment and polarization.

Macrophages are a key component of the tumor microenvironment and act as promoters of tumor cell invasion, migration, stromal degradation, and angiogenesis [52]. Both clinical and experimental studies have demonstrated that tumor-associated macrophages (TAMs) facilitate solid tumor metastasis through the release of various cytokines, including chemokines, inflammatory factors, and growth factors [53, 54]. Macrophages can be classified into two types, M1 and M2: M1 macrophages, activated by interferon gamma (IFN-γ) stimulation, exhibit antigen presentation, phagocytosis, cytotoxicity, and activation of the Th1 response through the secretion of IL-12, IL-1β, IL-6, and TNF-α. Consequently, M1 macrophages are considered to play a predominantly anti-tumor role [55–57]. In contrast, M2 macrophages are induced by IL-4 and IL-13, and this subpopulation secretes high levels of anti-inflammatory molecules, including IL-10, IL-4, arginase, and transforming growth factor-β (TGF-β), as well as vascular endothelial growth factors (VEGF-C, VEGF-A), which protect tumor cells from apoptosis and promote angiogenesis [57]. This primarily exerts a tumor-promoting function.

In the present study, to further explore the mechanisms related to M2-type macrophage polarization promoting the malignant progression of gastric cancer, we initially observed that VEGF secretion from M2-type macrophages induced by the culture supernatants of gastric cancer cells overexpressing Fra-1 was increased, whereas it was decreased in the group with Fra-1 knockdown. These results suggest that overexpression of Fra-1 in gastric cancer cells promotes the secretion of VEGF from M2-type macrophages in the microenvironment. Subsequently, we found that the culture supernatant-induced vascular endothelial cell tubule formation was enhanced in gastric cancer cells overexpressing Fra-1, whereas it was diminished after Fra-1 knockdown. This confirmed that Fra-1 promotes angiogenesis. We then confirmed that Fra-1 promotes angiogenesis by regulating the expression of HMGA2. In summary, in gastric cancer cells, Fra-1 promotes macrophage M2 polarization in the microenvironment and induces angiogenesis, thereby facilitating the malignant progression of gastric cancer cells through the regulation of HMGA2 expression (Fig. 6F). It is well established that M2-type macrophages promote tumor progression by suppressing the immune response in addition to regulating angiogenesis. However, whether the regulation of HMGA2 expression by Fra-1 in gastric cancer cells induces macrophage M2 polarization and affects the crosstalk between immune cells in the tumor microenvironment remains to be confirmed by further experiments. In addition, the subcutaneous xenograft model used in this study cannot fully reproduce the microenvironment of gastric cancer tumors. In the future, further studies employing genetically engineered mouse models with Fra-1 defects could better simulate the progression of gastric cancer. Additionally, by stimulating macrophages in these mice with LPS, the key roles of Fra-1 expression and macrophage function in the tumor microenvironment during gastric cancer progression could be further investigated.

In conclusion, this study demonstrates for the first time that Fra-1 enhances gastric cancer cell proliferation, invasion, and migration by binding to the HMGA2 promoter, thereby activating HMGA2 expression. This activation promotes CCL2 secretion in gastric cancer cells, induces M2-type macrophage polarization, and subsequently stimulates VEGF secretion to induce angiogenesis. Our findings partially elucidate the role of Fra-1 and its potential mechanisms in the development and progression of gastric cancer, which holds significant implications for the early diagnosis of gastric cancer, the identification of potential therapeutic targets, the management of advanced metastasis, and the prediction of patient prognosis.

## Materials and methods

### Cell culture

Human-derived gastric cancer cells HGC27 and AGS were cultured under the following conditions: 10% fetal bovine serum (FBS) (Procell, WuHan, China) supplemented with 1640 cell culture medium (Procell, WuHan, China), with cells grown in an adherent manner. Human umbilical vein endothelial cells (HUVECs) were cultured with 10% FBS (Procell, WuHan, China) and DMEM cell culture medium (Procell, WuHan, China), also using an adherent growth method. Human monocyte THP-1 cells were cultured in 10% FBS (Procell, WuHan, China) and 1640 cell culture medium (Procell, WuHan, China), with cells grown in suspension. All these cells are maintained at the Institute of Cancer Research, Central South University. The cell culture incubator settings were as follows: temperature at 37 °C and CO_2_ concentration of 5%.

### EdU cell proliferation assay

The treated cells were inoculated into appropriate well plates. EdU (10 mM) was diluted 1:1000 with cell culture solution to prepare a 1× working solution of EdU (10 μM), which was pre-warmed to 37 °C. This EdU working solution was then added to the well plates, and the cells were incubated for an additional 2 h. Following this incubation, the plates were removed, and the cells were washed 2–3 times using PBS. This washing process involved discarding the culture solution and fixing the cells. Subsequently, the cells were treated according to the instructions provided with the EdU Cell Proliferation Detection Kit (Beyotime, Shanghai, China). Finally, the cells were observed under a fluorescence microscope.

### Total RNA extraction by Trizol method

To the already cultured cells, add 1 mL of Trizol reagent. Thoroughly mix by pipetting, then transfer the mixture into a 1.5 mL EP (Eppendorf) tubule (ensure the tubule is RNase-free). Lysate the cells on ice for 10 min. Afterward, add 200 µL of chloroform, vortex to mix well, and then let the mixture sit on ice for 5 min. Centrifuge at 4 °C for 15 min at 12,000 rpm to separate the phases. Gently transfer the aqueous phase (upper layer) to a new tubule. Add an equal volume of isopropanol, mix well, and let it stand for 10 min. Afterward, discard the supernatant. Add 1 mL of 75% ethanol to wash the RNA, and repeat this wash step once more. Dry the RNA precipitate. After drying, resuspend the RNA pellet in 20 µL of DEPC-treated water. Determine the concentration of the RNA solution using a suitable spectrophotometer or other analytical method.

### RT-qPCR

The cDNA, primers, and Taq SYBR® Green qPCR Premix were mixed according to the manufacturer’s instructions for the Taq SYBR® Green qPCR Premix (iScience, Jiangsu, China). The mixture was then aliquoted into a plate, and the qPCR program was set up on the machine. For details on the primer sequences and concentrations, refer to Supplementary Table 1.

### Western blot

SDS-PAGE gels were prepared using the 10% ExpressCast PAGE Colour Gel Rapid Kit (NCMBIO, Suzhou, China). Once the gel was completely solidified, the comb teeth were quickly removed. A 50 µg protein sample was taken, and 5× loading buffer was added at a ratio of 4:1. The mixture was then placed in boiling water and heated at 100 °C for 5 min to denature the proteins. After denaturation, the sample was cooled down for loading onto the gel. Subsequently, electrophoretic separation was performed, followed by membrane transfer. After the transfer, a 5% blocking solution was applied to block non-specific binding sites. The membrane was then incubated with the primary antibody, followed by washing. Next, the membrane was incubated with the secondary antibody, and washed again. The ECL (enhanced chemiluminescence) luminescent solution was applied dropwise to the front side of the PVDF membrane. The membrane was then developed and imaged using a Western blot chemiluminescence imager. For details on the antibodies used, refer to Supplementary Table 2.

### Transwell

Using 24-well Transwell plates (pore size 8 μm; Corning, USA), Matrigel was diluted with serum-free medium and evenly spread on the bottom of the chambers. The chambers were then placed in a cytocentrifuge and left to solidify for 3–4 h. Subsequently, 3 × 10^4^ cells were suspended in 600 μL of basal medium containing 2% FBS and added to the upper chamber, while 800 μL of basal medium containing 20% FBS was placed in the lower chamber. After a 48 h incubation period, the Matrigel matrix and cells remaining in the upper chamber were removed using cotton swabs. The cells on the lower surface of the membrane were fixed with 4% paraformaldehyde and stained with 0.5% crystal violet. The cells were then observed under a microscope and photographed. All experiments were conducted in triplicate.

### Scratch healing test

Target cells were inoculated into six-well plates at a suitable density and treated according to the experimental requirements. Once the cells reached confluence (approximately 24–48 h later), a 10 μL pipette tip was used to create a vertical scratch in the cell monolayer within each well, following a straight edge. The cells were then washed three times with a cell washing solution to remove any debris. Fresh medium was added, and the width of the scratches was measured at the 0 h time point using a microscope. The plates were subsequently returned to the incubator for further cultivation. The width of the cell scratches was measured again at 24 and 48 h. The scratch widths at each time point were then analyzed using ImageJ software to assess cell migration.

### In vitro co-culture assay

Collect the supernatant from treated gastric cancer cells in a 15 mL centrifuge tubule. Centrifuge the tubule at 1000 × *g* for 10 min to pellet any cellular debris. Carefully aspirate the supernatant, and filter it through a 0.22 μm filter tip to remove any remaining particles. Transfer the filtered supernatant to a new 15 mL centrifuge tubule to create the co-culture conditioned medium. This conditioned medium can be stored at 4 °C for up to 1 week or at −80 °C for up to 6 months. For the co-culture, take 30% to 60% of the conditioned medium and add it to the macrophage culture. Incubate the macrophages with the conditioned medium for 24 h. After the incubation period, you can proceed with the relevant experimental tests.

### Flow cytometry

Centrifuge the single cell suspension at 1000 rpm for 5 min. Remove the supernatant, and add 1 mL of PBS (phosphate-buffered saline) to the cell pellet. Gently mix and wash the cells twice to remove any remaining supernatant. Next, prepare the following groups: a blank group, a single standard group, a control group, and an experimental group. To each cell sample, add 400 μL of a membrane-breaking agent and mix well to ensure even distribution. Divide the mixture into 100 μL aliquots for each of the groups mentioned above. Add an appropriate amount of the specific antibody to the corresponding group, and incubate at room temperature, protected from light, for 30 min. After incubation, wash each group twice with PBS, mixing gently to ensure thorough washing. Finally, proceed with detection on the appropriate machine, such as a flow cytometer or plate reader, depending on the assay being performed.

### Co-IP

Treated cells were lysed with IP lysis buffer for 30 min on ice. The lysate was then centrifuged at 14,000 × *g* for 15 min at 4 °C, and the supernatant was carefully transferred to a new centrifuge tubule. A small aliquot (40 μL) of the lysate was taken as the input control for Western blotting analysis. Next, prepare two sets of magnetic beads (50 μL each): one set is coated with the specific antibody for the target protein, and the other is used to bind and remove non-specific proteins. Add 500 μL of each lysed cell sample to the corresponding magnetic beads and incubate at 4 °C, with rotation, overnight. The following day, prepare another two sets of magnetic beads (50 μL each): one with the antibody and the other with non-specific proteins removed. Add 500 μL of the lysed cells to the corresponding magnetic beads, and rotate the mixture at 4 °C overnight. After incubation, wash the beads three times with the lysis buffer to remove any unbound material. Add SDS sample loading buffer to the beads, gently resuspend the beads-antibody-antigen complexes, and heat them at 100 °C for 8 min to denature the proteins. Finally, perform SDS-PAGE electrophoresis to separate the proteins, followed by Western blotting for analysis.

### EMSA

Firstly, the target probe was designed, biotin-labeled, and annealed to complete the biotin-labeled probe. Subsequently, a transcription factor Fra-1 overexpression plasmid was transfected into gastric cancer cells. After 48 h, nuclear proteins were extracted and set aside. Next, 6% nondenaturing polyacrylamide gels were prepared using the following components: 7.15 ml of ultrapure water, 2 ml of 30% polyacrylamide solution (Solarbio, A1011), 1 ml of 5× TBE (Solarbio, T1051), 175 μl of 80% glycerol (Solarbio, G8190), 75 μl of 10% APS (Solarbio, A1030), and 8 μl of TEMED (Solarbio, T8090). After resting at 37 °C for 30 min, pre-electrophoresis was performed at 100 V for 30 min. Next, the sample reaction solution and control reaction solution were prepared using the chemiluminescence EMSA kit (Beyotime, GS009). The samples were loaded and electrophoresed at 100 V for 45 min. Subsequently, the gel was transferred to a nylon membrane (Solarbio, YA1760) for 45 min. The nylon membrane was then blocked, sealed, equilibrated, and finally imaged using chemiluminescence.

### ChIP

The ChIP assay kit (Beyotime, Wuhan, China) was used according to the instructions provided by the manufacturer. Firstly, the treated cells were crosslinked and broken by ultrasound, then immunoprecipitated and uncrosslinked, and finally the products were purified and detected by RT-qPCR. The kit information is shown in Supplementary Table 3.

### Dual luciferase reporter assay

Luciferase activity was measured using the Dual Luciferase Reporter Gene Assay Kit (Uelandy, Suzhou, China), following the manufacturer’s instructions. The treated cells were lysed to ensure complete cell disruption. The lysed cells were then assayed in an opaque 96-well plate to protect the luciferase reaction from light interference.

### Tubule formation experiment

Vascular endothelial cells in the logarithmic growth phase were trypsinized to detach them from the culture surface. The cells were then counted and resuspended to a concentration of 3000 cells/mL. A volume of 100 μL of this cell suspension was added to each well of a 96-well plate that had been pre-coated with Matrigel to promote adhesion. The inoculated cells were cultured in a cell incubator for 4–6 h at 37 °C with 5% CO_2_ to allow for the formation of lumen-like structures. Following the incubation period, the cells were observed under a microscope to assess the formation of the lumen. Images of the structures were captured, and the results were quantitatively analyzed using ImageJ software for a more detailed evaluation.

### Subcutaneous tumor formation in nude mice

Four-week-old female BALB/c nude mice were randomly divided into four groups, each consisting of five mice. They were housed in the Department of Zoology, Xiangya School of Medicine, Central South University, and acclimated for 2 weeks before subsequent experiments. Gastric cancer cells HGC27, which were stably overexpressing Fra-1, and the negative control cells, were trypsinized, washed three times with saline, counted, and then resuspended in saline to a concentration of 1 × 10^7^ cells/mL. A volume of 200 μL (corresponding to 5 × 10^6^ cells) of the cell suspension was injected into the right axillary region of each nude mouse. Tumor formation was observed every four days, and the body weights of the nude mice were recorded. After tumor formation, the nude mice were injected intraperitoneally with INCB3344 at a dosage of 10 mg/kg. After 3–4 weeks, when the tumors had reached a suitable size, the nude mice were euthanized, and the subcutaneous tumor tissues were excised. The tumors were weighed, photographed, washed three times in 1× PBS, fixed in 4% paraformaldehyde at room temperature, and then prepared for immunohistochemistry for subsequent experiments.

### Statistical analysis

Two-group comparisons were analyzed using the Student’s *t* test, while multiple-group comparisons were analyzed using analysis of variance (ANOVA). Each experiment was repeated more than three times, and the data are presented as the mean ± standard deviation. All statistical analyses are described in the legend of each figure. Correlations between groups were assessed using GraphPad Prism 9 software.

All data were analyzed to determine significant differences, with the following notation: p < 0.05 is denoted by *; p < 0.01 is denoted by **; p < 0.001 is denoted by ***; and p < 0.0001 is denoted by ****; Non-significant results are indicated as “ns”. Statistical plots were generated, and the data counts were performed using GraphPad Prism 9 software.

## Supplementary information

Supplementary Figure Legends

Supplementary Table 1

Supplementary Table 2

Supplementary Table 3

Supplementary Table 4

Supplementary Figure 1

Supplementary Figure 2

Supplementary Figure 3

Supplementary Figure 4

Original Western Blots

## Data Availability

Original data are available upon request. The full length uncropped original western blots are shown in the “Supplementary Material”.
