# Supplementary material for: Fra-1 promotes gastric cancer progression by regulating macrophage polarization and transcriptionally activating HMGA2 expression
Source: Cell Death Discov. 2025 Oct 6;11:433. doi: 10.1038/s41420-025-02724-1 (PMC12500915; doi:10.1038/s41420-025-02724-1)
Supplement: Supplementary file 1 — Supplementary Figure Legends [file 41420_2025_2724_MOESM1_ESM.docx]

**Supplementary Figure Legends：**

**Supplementary Figure 1 Fra-1 enhances the proliferative, invasive, and migratory abilities of gastric cancer cells**

1. B) Verification of Fra-1 overexpression or knockdown in gastric cancer cells through RT-qPCR analysis. (C-D) Co-Immunoprecipitation (Co-IP) assay in gastric cancer cells to confirm the interaction between Fra-1 and HMGA2 proteins. (E-F) Assessment of the effects of Fra-1 knockdown, either alone or in combination with HMGA2 knockdown, on the proliferative capacity of gastric cancer cells using an EdU cell proliferation assay combined with flow cytometry. (G-H) Evaluation of the invasion ability of gastric cancer cells AGS following Fra-1 overexpression or knockdown, either alone or in combination with HMGA2 knockdown, using a Transwell cell invasion assay. (I-K) Determination of the migratory ability of gastric cancer cells after Fra-1 overexpression or knockdown, either alone or in combination with HMGA2 knockdown, using a scratch healing assay. All experiments were conducted in three or more independent replicates, and the data presented are from representative individual experiments. "ns" indicates no significant difference; "*" indicates p < 0.05; "**" indicates p < 0.01; "***" indicates p < 0.001; "****" indicates p < 0.0001.

**Supplementary Figure 2 Fra-1 binds specifically to the second site of the HMGA2 promoter region**

(A**-**B) The EMSA experiment was used to detect whether Fra-1 directly binds to the HMGA2 promoter region in gastric cancer cells HGC27/AGS. Probe (952-964): Biotin-labeled probe for the second binding site of the HMGA2 promoter; Cold Probe: Unlabeled probe for the second binding site of the HMGA2 promoter; Nuclear Protein: Nuclear protein containing the transcription factor Fra-1.

**Supplementary Figure 3 Fra-1 regulation of HMGA2 expression promotes CCL2 binding to CCR2 and induces macrophage M2 polarization**

1. B) In vitro co-culture experiments were conducted to detect the effects of Fra-1 overexpression and Fra-1 overexpression with concurrent HMGA2 knockdown in gastric cancer cells HGC27 on macrophage M1 type and polarization ratio using flow cytometry. (C-H) The impact of Fra-1 on the secretion of M2-type macrophage-associated cytokines TGF-β, IL-10, and Arg-1 by modulating HMGA2 expression in gastric cancer cells HGC27 was assessed using in vitro co-cultivation experiments and an ELISA assay kit. (I) Conditioned medium from gastric cancer cells HGC27 overexpressing Fra-1 was prepared and co-cultured with M2-type macrophages. The co-localization of CCL2 secreted by gastric cancer cells and CCR2 expressed by macrophages was detected using laser confocal microscopy. (J) Gastric cancer cells HGC27 overexpressing Fra-1 were used to prepare conditioned medium, which was co-cultured with M2-type macrophages in the presence or absence of the CCR2 antagonist INCB3344. The co-localization of CCL2 and CCR2 was detected using laser confocal microscopy. (K-L) Conditioned medium from gastric cancer cells overexpressing Fra-1 was co-cultured with successfully induced M2-type macrophages, with or without the addition of the CCR2 antagonist INCB3344. The secretion of IL-10, a marker cytokine of M2-type macrophages, was detected using an ELISA assay kit. All experiments were performed in three or more independent replicates, and the data shown are from representative individual experiments. "ns" indicates no significant difference; "*" indicates p < 0.05; "**" indicates p < 0.01; "***" indicates p < 0.001; "****" indicates p < 0.0001.

**Supplementary Figure 4 Fra-1 induces macrophage M2 polarization and promotes the secretion of vascular growth factor VEGF.**

(A) RNA-seq was performed on M0 and M2 macrophages, and the heat map showed the differential factors related to angiogenesis in M2 macrophages. The top three factors were focused according to the upregulation ratio and P value: MMP12, MMP7, and VEGF. (B-C) Overexpression of Fra-1 in gastric cancer cells, and collecting conditioned medium for co-cultivation with M0 macrophages in vitro, and detecting the expression of MMP12, MMP7 and VEGF in macrophages by RT-qPCR. (D-E) Overexpression of Fra-1 in gastric cancer cells, overexpression of Fra-1 and knockdown of HMGA2 expression, and collecting conditioned medium for in vitro co-cultivation with M0 macrophages were used to detect the expression of MMP12, MMP7 and VEGF in macrophages by RT-qPCR.
