# Supplementary material for: Fra-1 promotes gastric cancer progression by regulating macrophage polarization and transcriptionally activating HMGA2 expression
Source: Cell Death Discov. 2025 Oct 6;11:433. doi: 10.1038/s41420-025-02724-1 (PMC12500915; doi:10.1038/s41420-025-02724-1)
Supplement: Supplementary file 2 — Supplementary Table 1 [file 41420_2025_2724_MOESM2_ESM.docx]

**Supplementary Table 1 Primer sequence**

| Gene name | The upstream and downstream primer sequences |
| --- | --- |
| Fra-1 | Forward 5‘-CAGTGGATGGTACAGCCTCATTTC-3’  Reverse 5‘-GCAGTCTCCTGTTCACAAGGC -3’ |
| ITSN2 | Forward 5‘-TCAAGAAGGTGGTGAAGCAGG -3’  Reverse 5‘-TCAAAGGTGGAGGAGTGGGT-3’ |
| IQCB1 | Forward 5‘ACACCAGGTATTTTGATGAGGAG-3’  Reverse 5‘TCAGGCCGTGCCGCTGGCCGAGTAG-3 |
| MED18 | Forward 5‘AGCTGGTTTGGATCTTCGGA-3’  Reverse 5‘CAGGTCATCCCCAGAGTTGT-3’ |
| CYP26B1 | Forward 5‘AGTTCATGTCACGCTGGGTA-3’  Reverse 5‘CAGCTTCAGGTCTCCTTGGA-3’ |
| HMGA2 | Forward 5‘GCAACCCTTCTTTGACAACATTTTT-3’  Reverse 5‘ATTTCTTCTCTCAGACGCTCTCC-3’ |
| TGF-β | Forward 5‘-AACTTCCTAGTCGGATTGC-3  Reverse 5‘-TTGGTGGTGGTCTCCTTA-3’ |
| IL-10 | Forward 5‘-AACTTCCTAGTCGGATTGC-3  Reverse 5‘-TTGGTGGTGGTCTCCTTA-3’ |
| Arg-1 | Forward 5‘-AACTTCCTAGTCGGATTGC-3  Reverse 5‘-TTGGTGGTGGTCTCCTTA-3’ |
| MMP7 | Forward 5‘-TGGAATGTTAAACTCCCGCG-3  Reverse 5‘-TGCCCCACATGTTTAAAGCC-3’ |
| MMP12 | Forward 5‘-GTTCCTCACTGCTGTTCACG-3  Reverse 5‘-GCGTTGGTTCTCTTTTGGGT-3’ |
| VEGF | Forward 5‘-CGGTATAAGTCCTGGAGCGT-3  Reverse 5‘-TTTAACTCAAGCTGCCTCGC-3’ |
