# Supplementary material for: Fra-1 promotes gastric cancer progression by regulating macrophage polarization and transcriptionally activating HMGA2 expression
Source: Cell Death Discov. 2025 Oct 6;11:433. doi: 10.1038/s41420-025-02724-1 (PMC12500915; doi:10.1038/s41420-025-02724-1)
Supplement: Supplementary file 3 — Supplementary Table 2 [file 41420_2025_2724_MOESM3_ESM.docx]

**Supplementary Table 2 Antibody information**

| Antibody name | Company | Antibody dilutions | catalog numbers |
| --- | --- | --- | --- |
| Fra-1 | ImmunoWay | 1:1000 | YT1772 |
| CCL2 | HuaBio | 1:1000 | HA500042 |
| CCR2 | Abiowell | 1:1000 | AWA10339 |
| Flag | ImunnoWay | 1:5000 | YM3808 |
| IgG | Santa Cruz  Biotechnology | 2μg | sc-52336 |
| GAPDH | ImmunoWay | 1:5000 | YM8394 |
| HMGA2 | HuaBio | 1:1000 | HA722295 |
| CD11b-PerCP-cy5.5 | Biolegend | 1:50 | 982610 |
| CD86-PE-Cy7 | Biolegend | 1:50 | 374210 |
| CD163-APC | Biolegend | 1:50 | 326510 |
| Fra-1-ChIP | Santa Cruz  Biotechnology | 2μg | sc-28310 |
| Ki67 | ImmunoWay | 1:1000 | YM8189 |
