# Supplementary material for: Fra-1 promotes gastric cancer progression by regulating macrophage polarization and transcriptionally activating HMGA2 expression
Source: Cell Death Discov. 2025 Oct 6;11:433. doi: 10.1038/s41420-025-02724-1 (PMC12500915; doi:10.1038/s41420-025-02724-1)
Supplement: Supplementary file 4 — Supplementary Table 3 [file 41420_2025_2724_MOESM4_ESM.docx]

**Supplementary Table 3 Reagent information**

| Name | Product Code | Company |
| --- | --- | --- |
| INCB3344 | HY-12320 | MCE |
| RPMI-1640 | PMI50110 | Procell |
| DMEM | PMI50220 | Procell |
| [ChIP Assay Kit](https://www.beyotime.com/product/P2078.htm) | P2078 | Beyotime |
| FBS | 164210-50 | Procell |
| TGF-β ELISA Kit | ELK1827 | Elkbiotech |
| IL-10 ELISA Kit | ELK1147 | Elkbiotech |
| Arg-1 ELISA Kit | ELK1790 | Elkbiotech |
| CCL2 ELISA Kit | E-EL-M3001 | Elabscience |
| VEGF ELISA Kit | ELK1008 | Elkbiotech |
| [BeyoClick™ EdU-488](https://www.beyotime.com/product/C0071L.htm) | C0071L | Beyotime |
| Taq SYBR® Green qPCR Premix | EG20117M | iScience |
| [Dualucif® Firefly&Renilla Assay Kit](http://www.uelandy.com/productDe_19.html" \o "Dualucif<sup>®</sup> Firefly & Renilla Assay Kit（双萤光素酶报告基因检测试剂盒）) | F6075M | UElandy |
| Chemiluminescence EMSA kit | GS009 | Beyotime |
