# Supplementary material for: Fra-1 promotes gastric cancer progression by regulating macrophage polarization and transcriptionally activating HMGA2 expression
Source: Cell Death Discov. 2025 Oct 6;11:433. doi: 10.1038/s41420-025-02724-1 (PMC12500915; doi:10.1038/s41420-025-02724-1)
Supplement: Supplementary file 5 — Supplementary Table 4 [file 41420_2025_2724_MOESM5_ESM.docx]

**Supplementary Table 4 The JASPAR database predicts Fra-1 binding sites to the HMGA2 promoter**

| Matrix ID | Name | Score | Start | End | Strand | Predicted sequence |
| --- | --- | --- | --- | --- | --- | --- |
| MA0477.1 | FOSL1 | 6.2319527 | 193 | 203 | + | AGTGACTCCCC |
| MA0477.1 | FOSL1 | 4.3866186 | 193 | 203 | - | GGGGAGTCACT |
| MA0477.1 | FOSL1 | 6.4889703 | 952 | 964 | + | ACATAACTCAGAA |
| MA0477.1 | FOSL1 | 3.8837733 | 953 | 963 | - | TCTGAGTTATG |
| MA0477.1 | FOSL1 | 5.651597 | 192 | 204 | + | CAGTGACTCCCCT |
