# Supplementary figures and images for: Fra-1 promotes gastric cancer progression by regulating macrophage polarization and transcriptionally activating HMGA2 expression
Source: Cell Death Discov. 2025 Oct 6;11:433. doi: 10.1038/s41420-025-02724-1 (PMC12500915; doi:10.1038/s41420-025-02724-1)

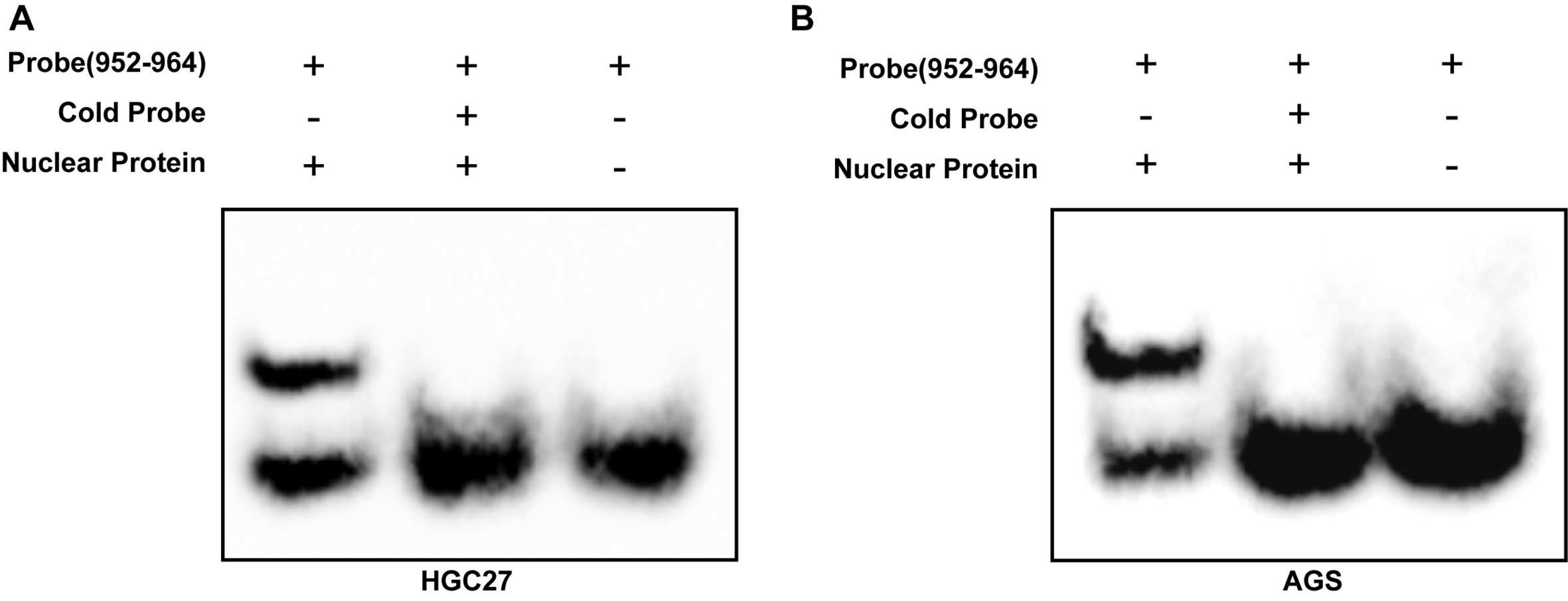

Supplement: Supplementary file 7 — Supplementary Figure 2 [file 41420_2025_2724_MOESM7_ESM.tif]

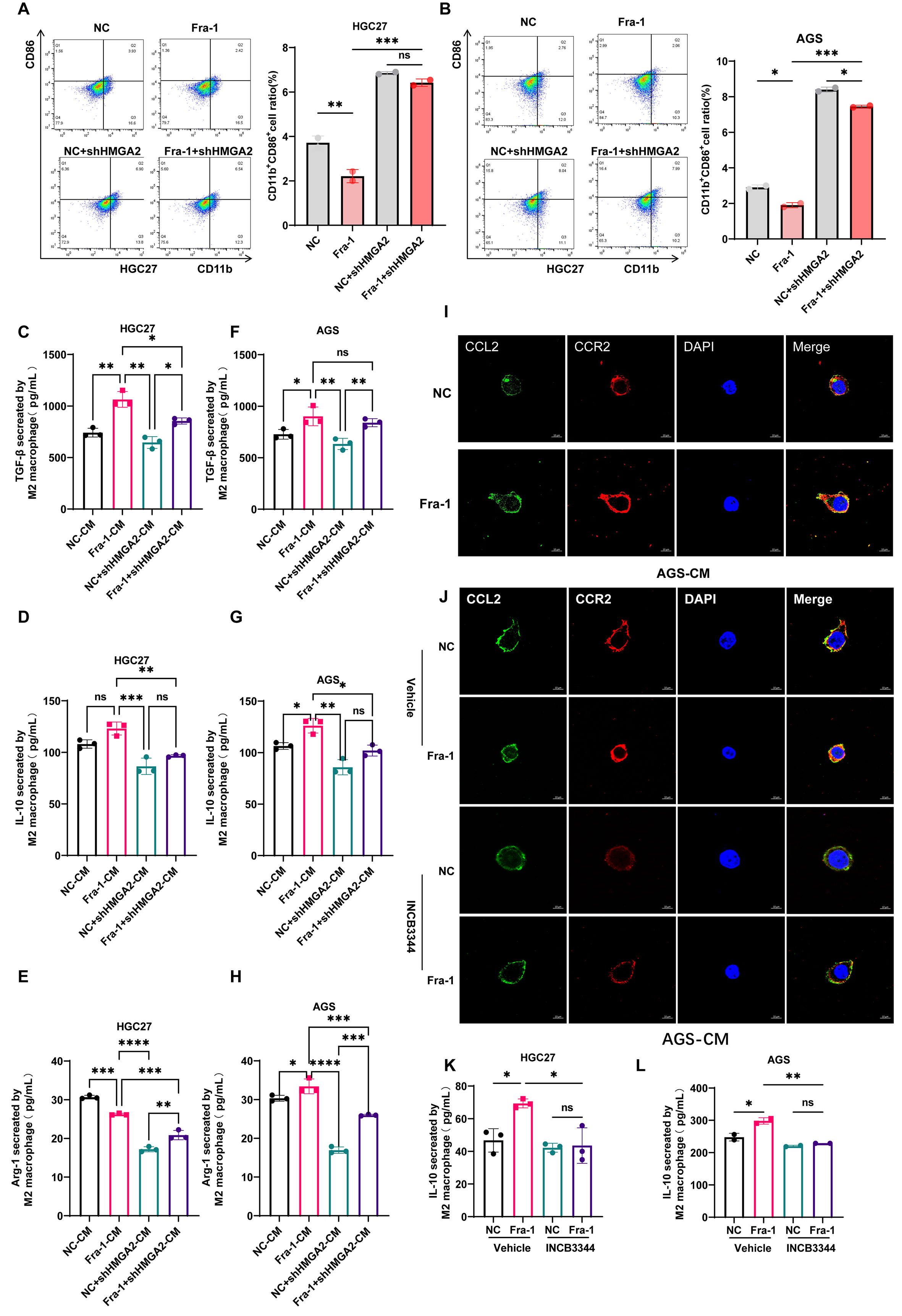

Supplement: Supplementary file 8 — Supplementary Figure 3 [file 41420_2025_2724_MOESM8_ESM.tif]

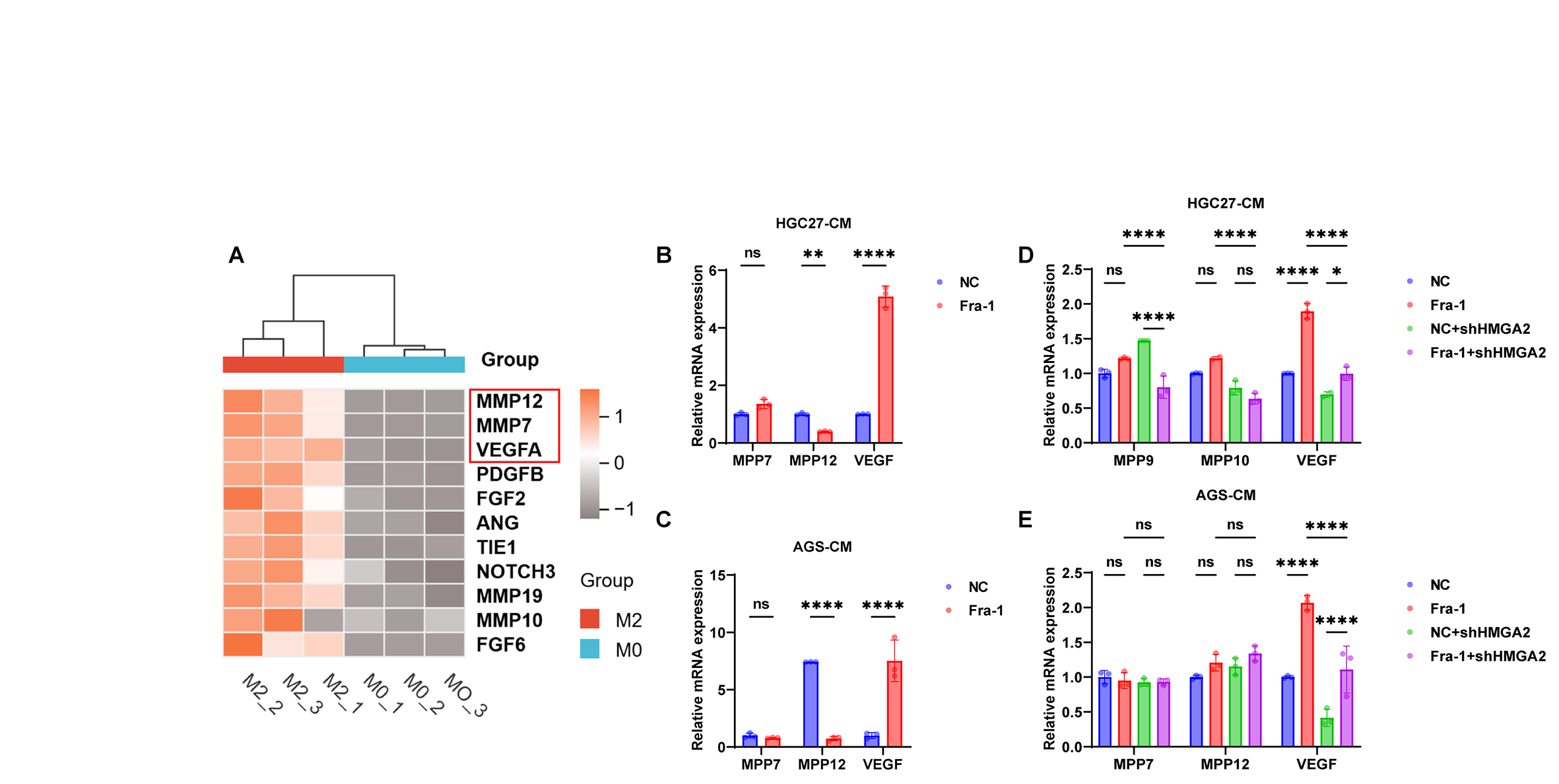

Supplement: Supplementary file 9 — Supplementary Figure 4 [file 41420_2025_2724_MOESM9_ESM.tif]
