# Supplementary material for: Fra-1 promotes gastric cancer progression by regulating macrophage polarization and transcriptionally activating HMGA2 expression
Source: Cell Death Discov. 2025 Oct 6;11:433. doi: 10.1038/s41420-025-02724-1 (PMC12500915; doi:10.1038/s41420-025-02724-1)

Figure 2G-Fra-1-1

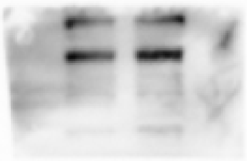

Figure 2G-Fra-1-2

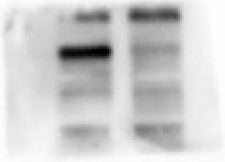

Figure 2G-HMGA2-1

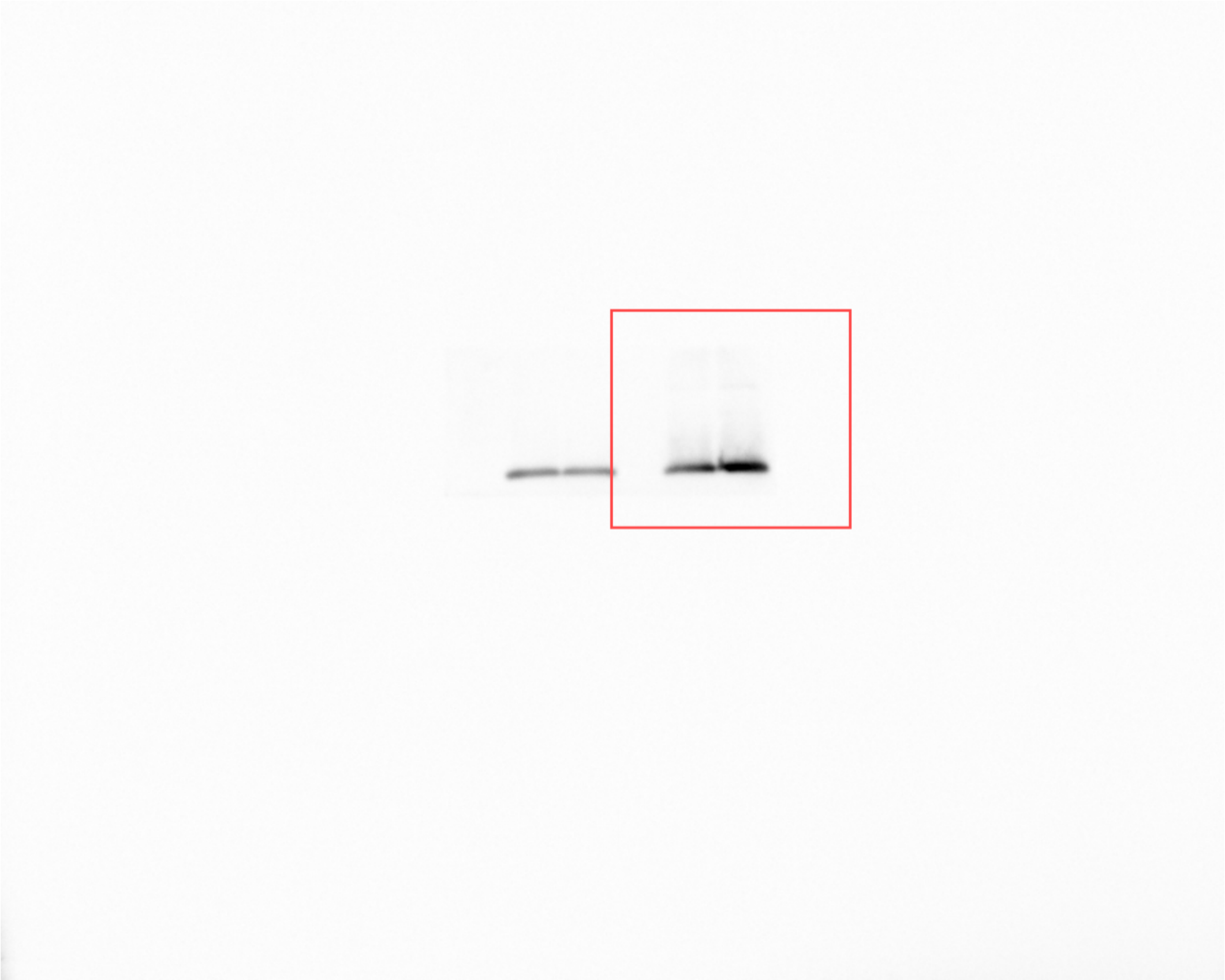

Figure 2G-HMGA2-2

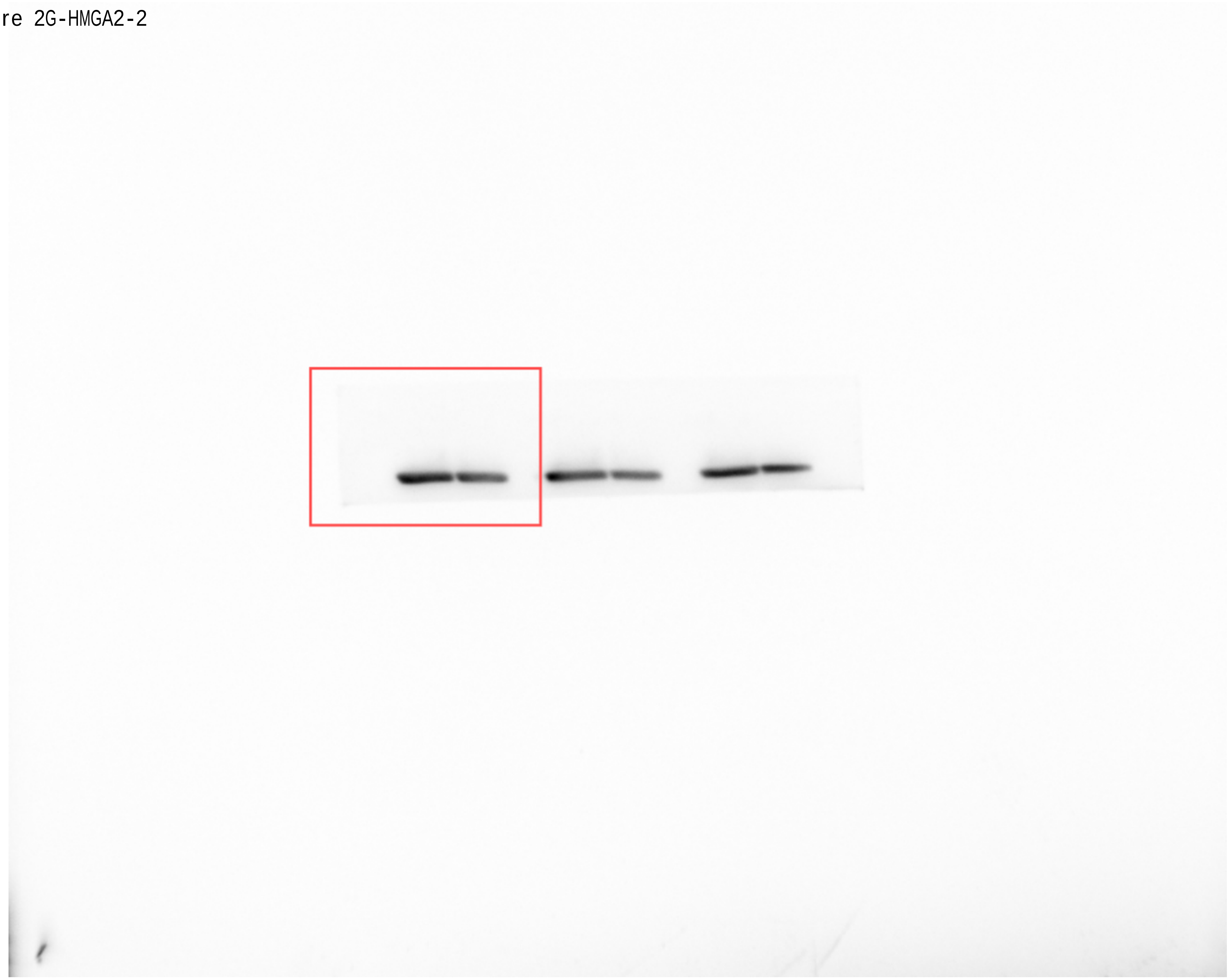

Figure 2G-GAPDH

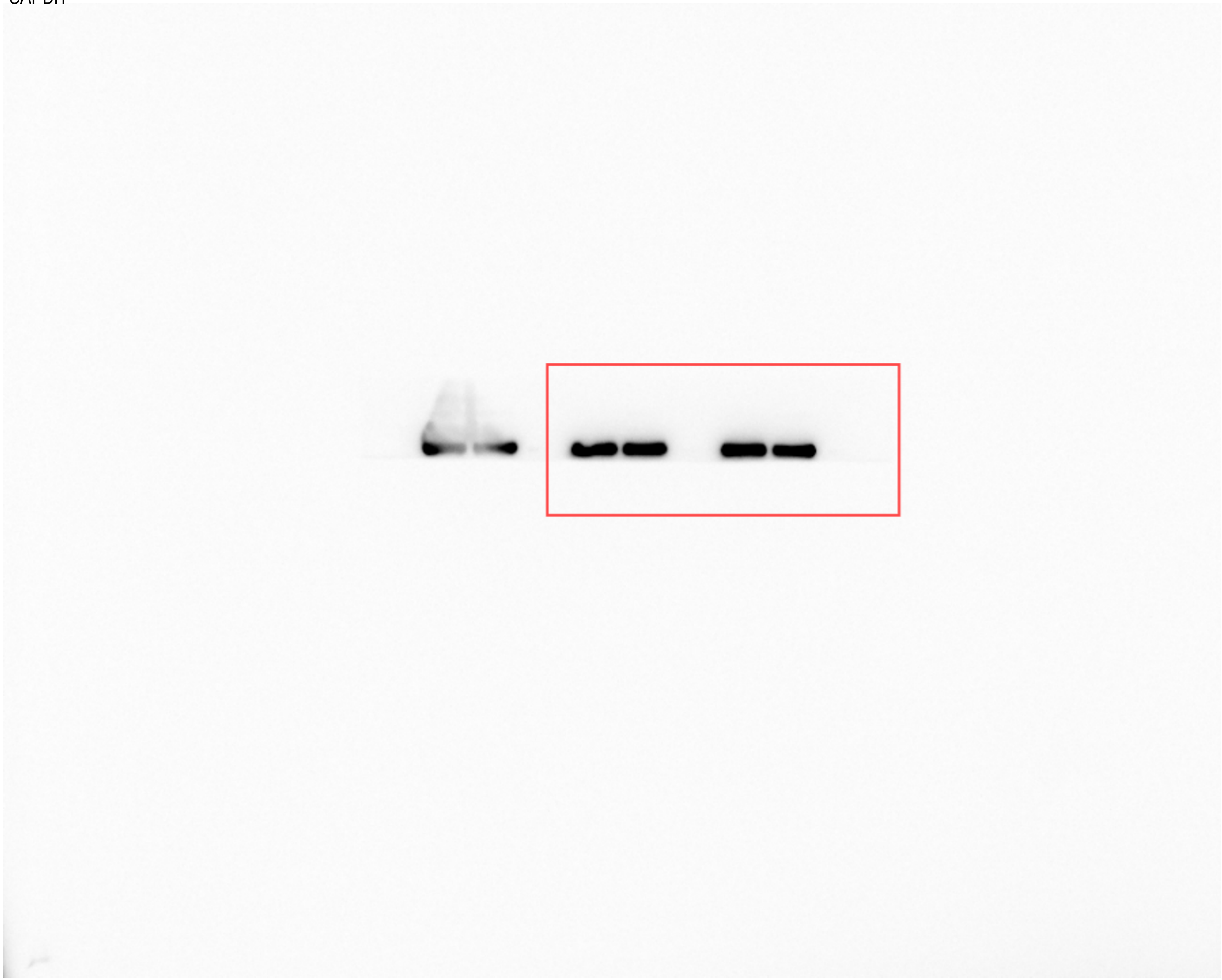

Figure 2H-Fra-1-1

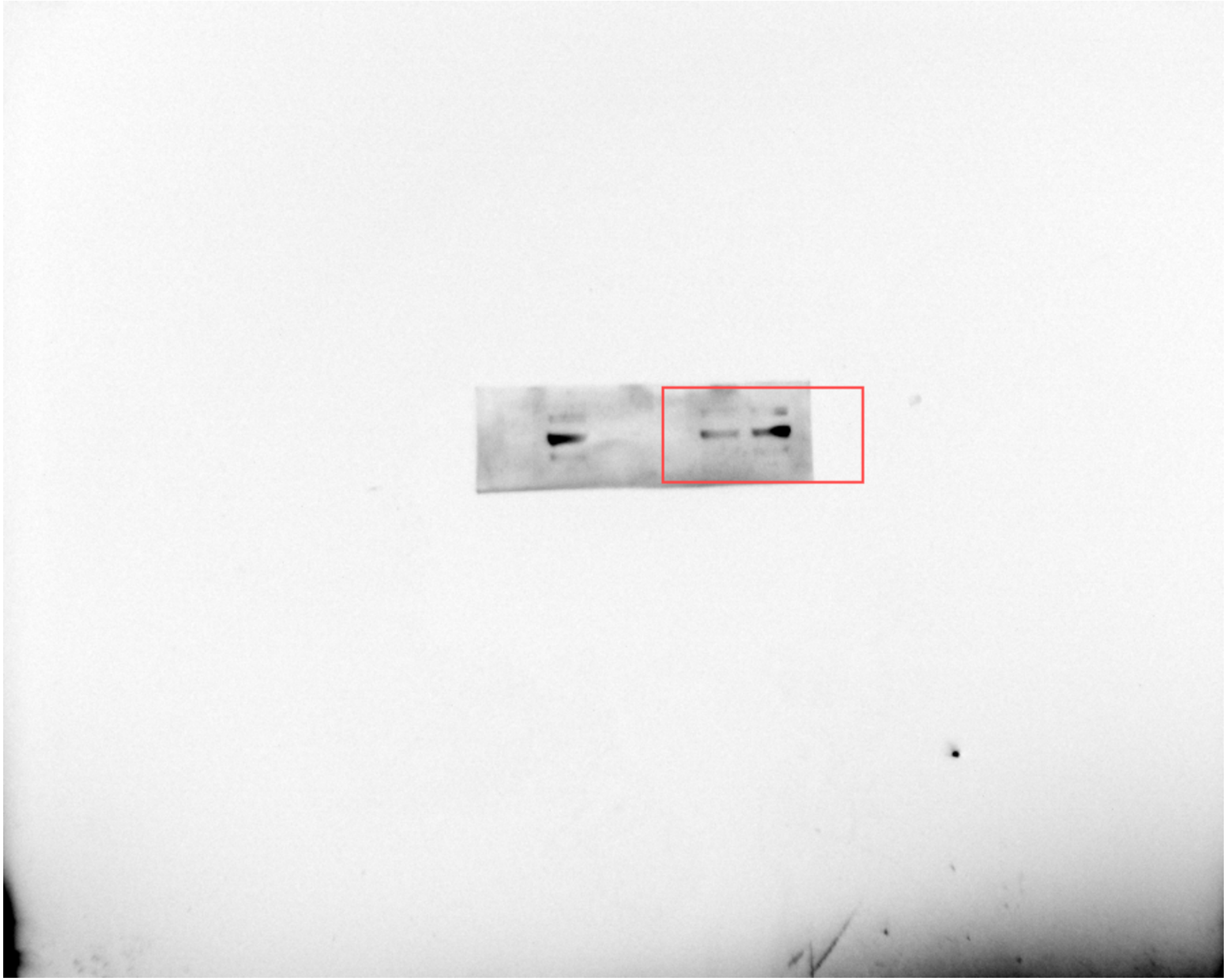

Figure 2H-Fra-1-2

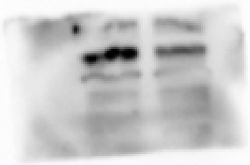

Figure 2H-HMGA2-1

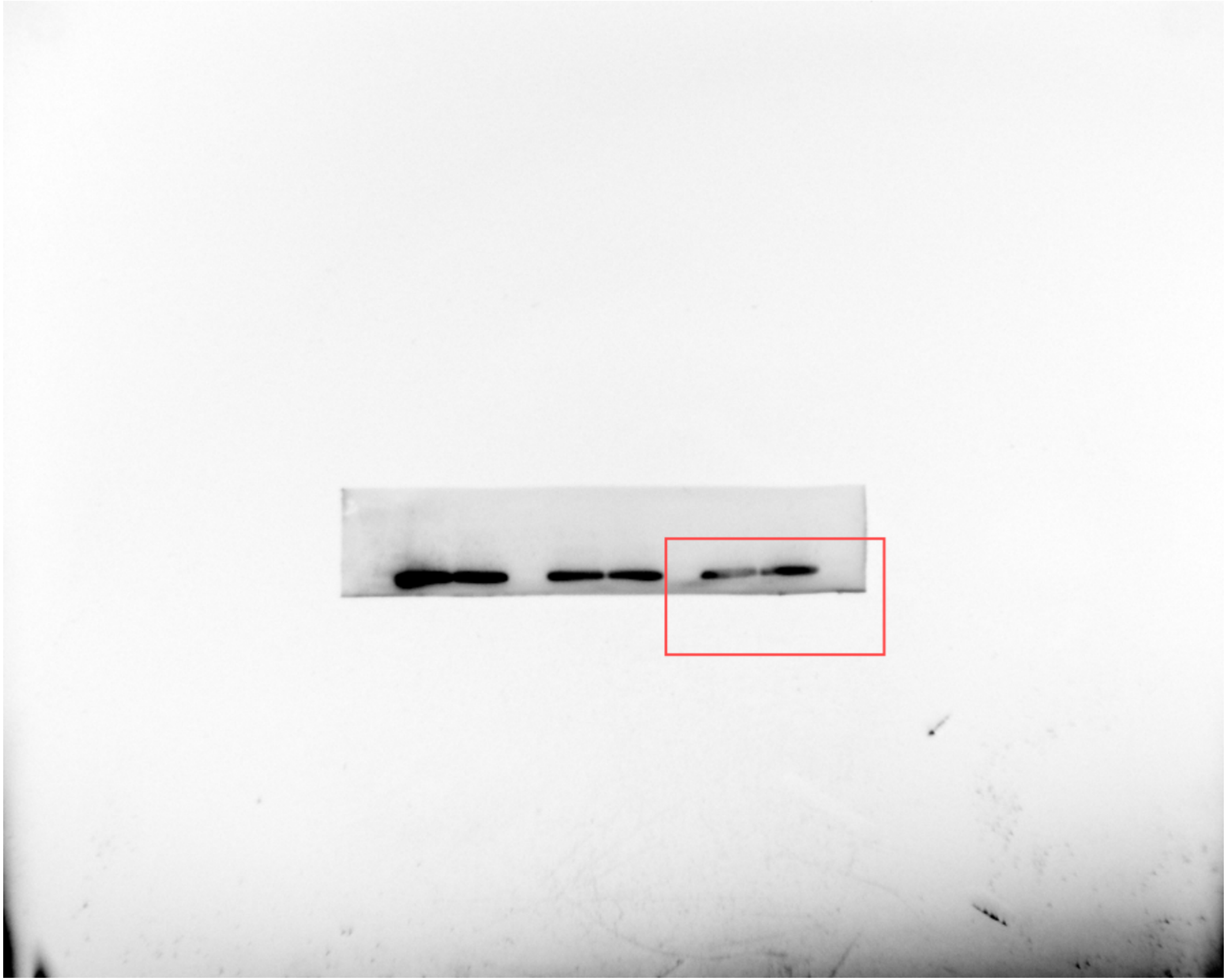

Figure 2H-HMGA2-2

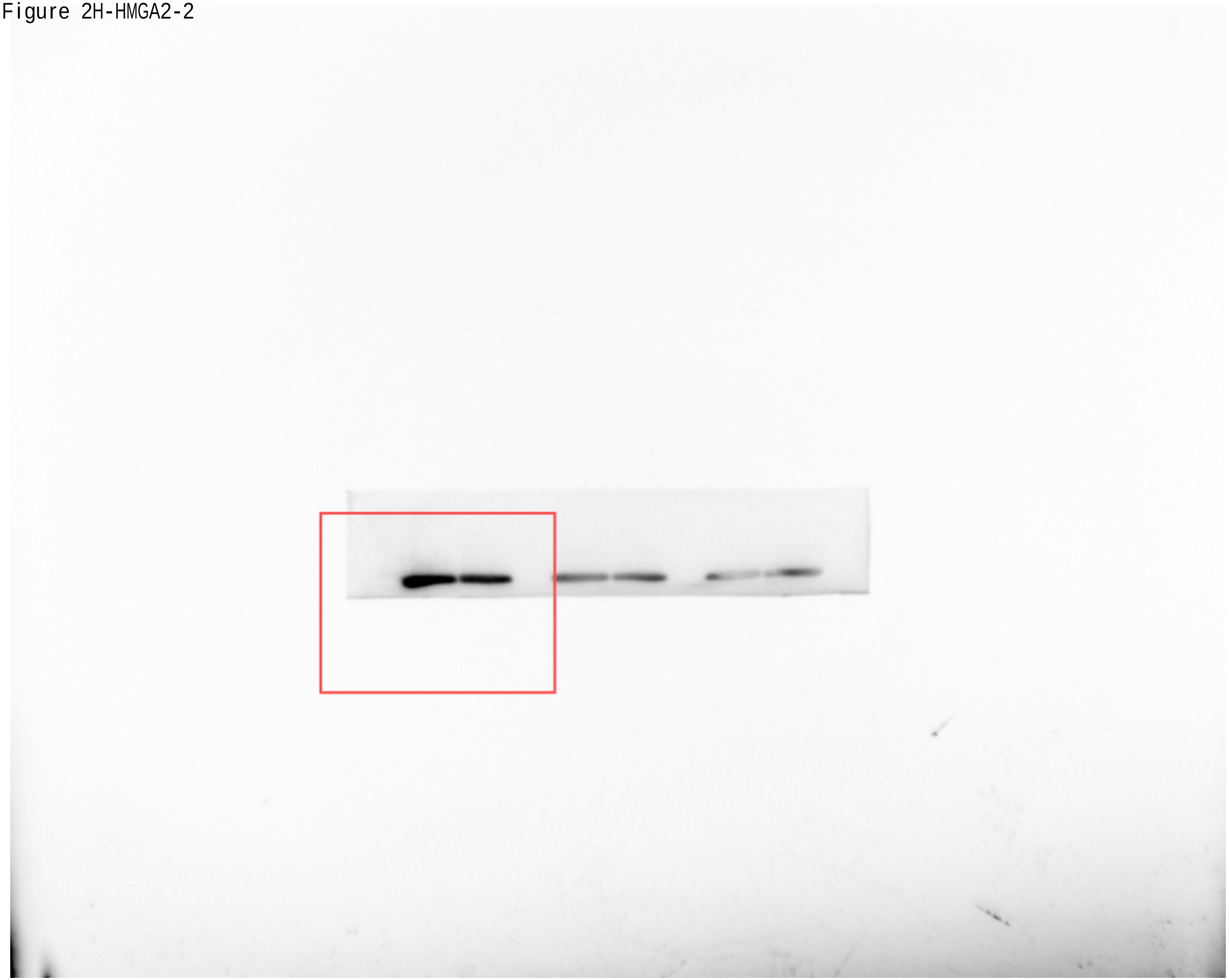

Figure 2H-GAPDH

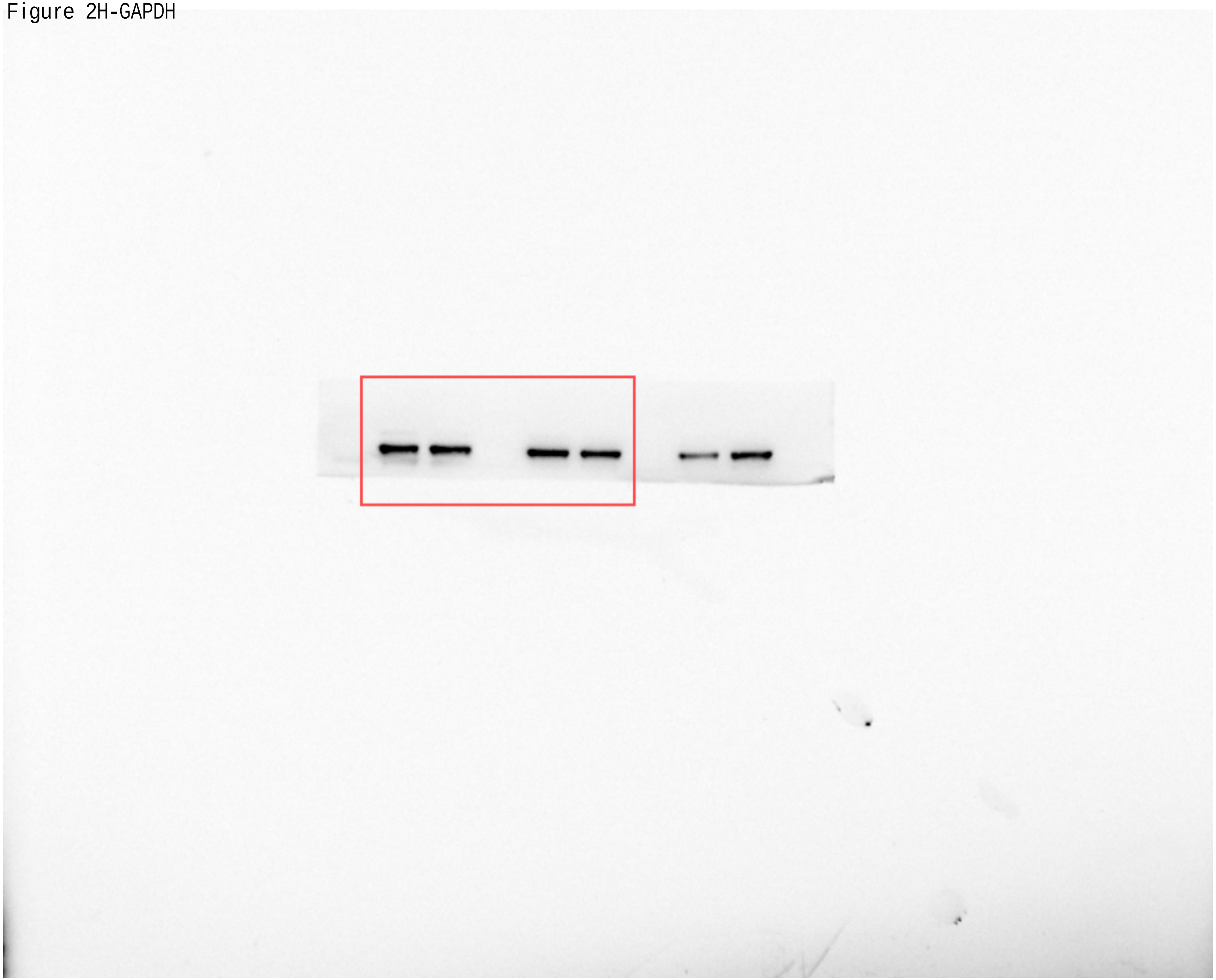

Figure 4G-CCL2-1

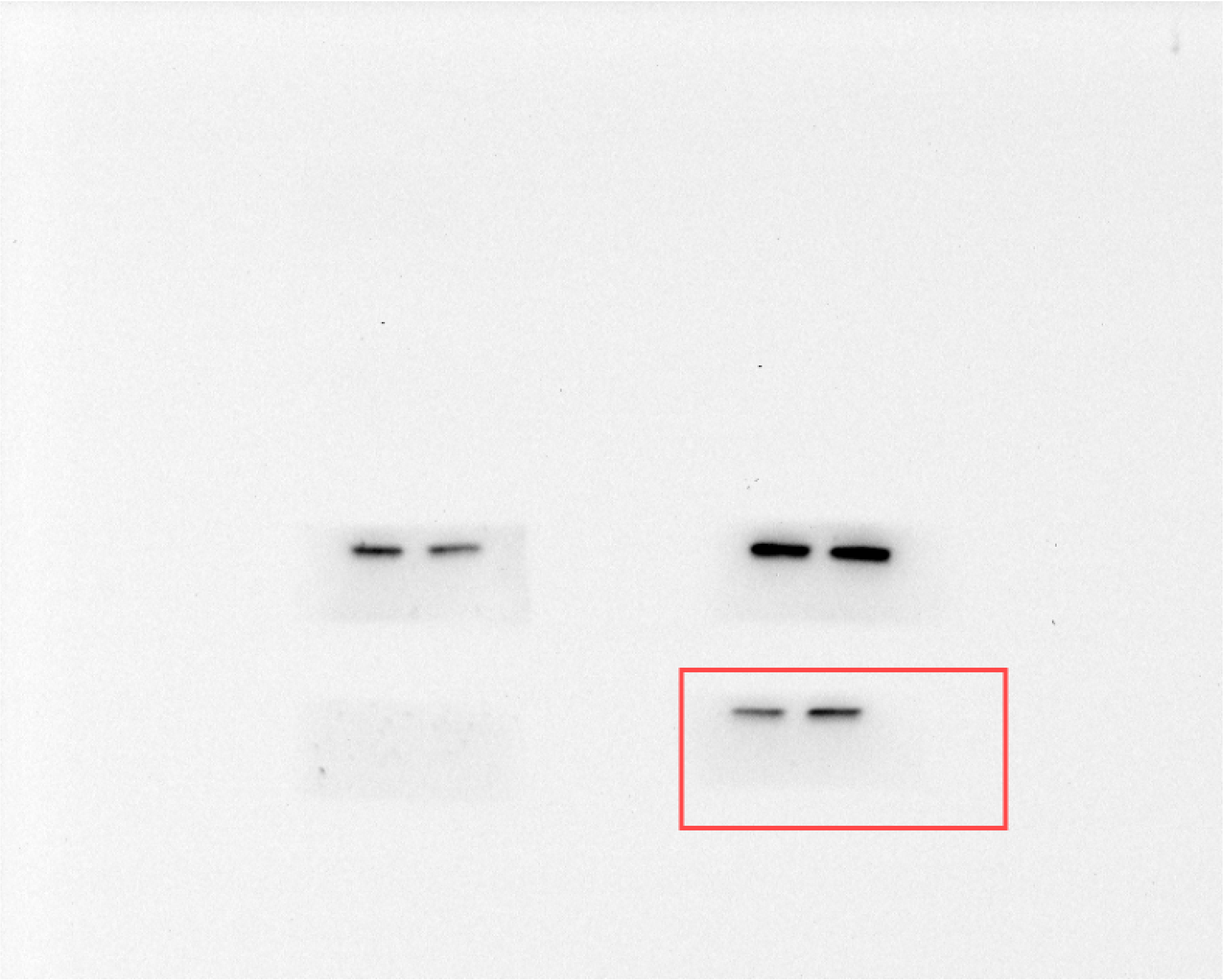

Figure 4G-CCL2-2

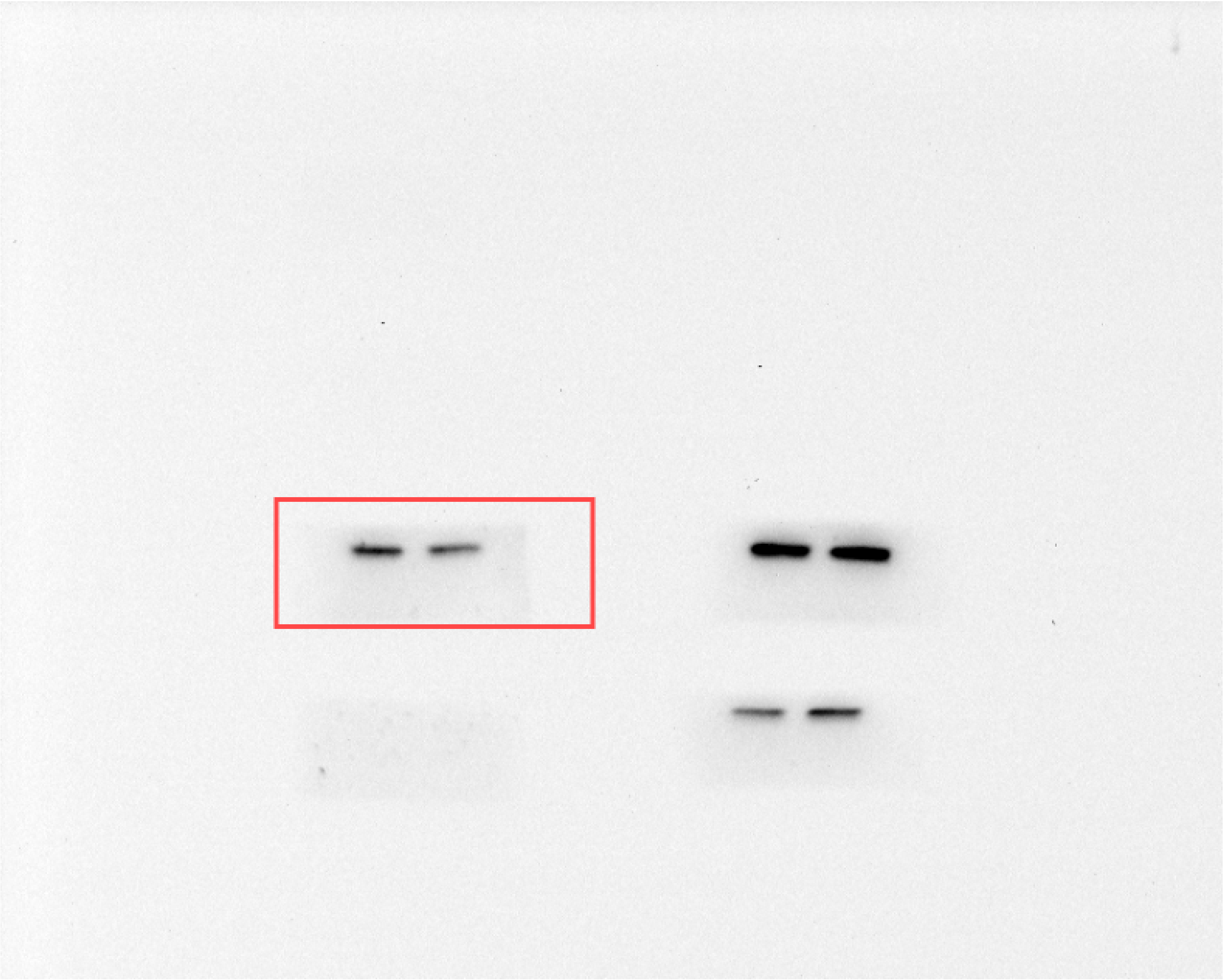

Figure 4G-HMGA2-2

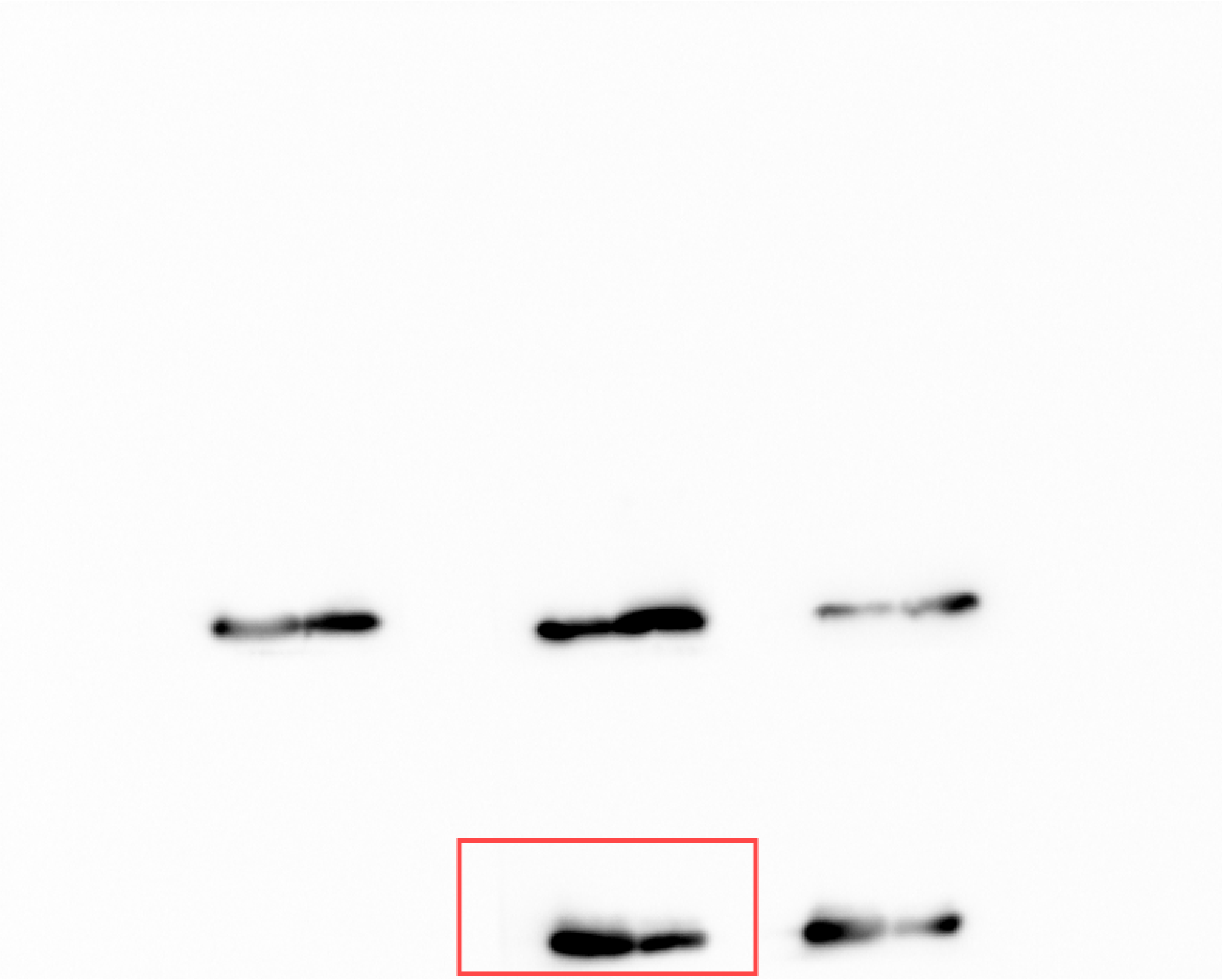

Figure 4G-HMGA2-1

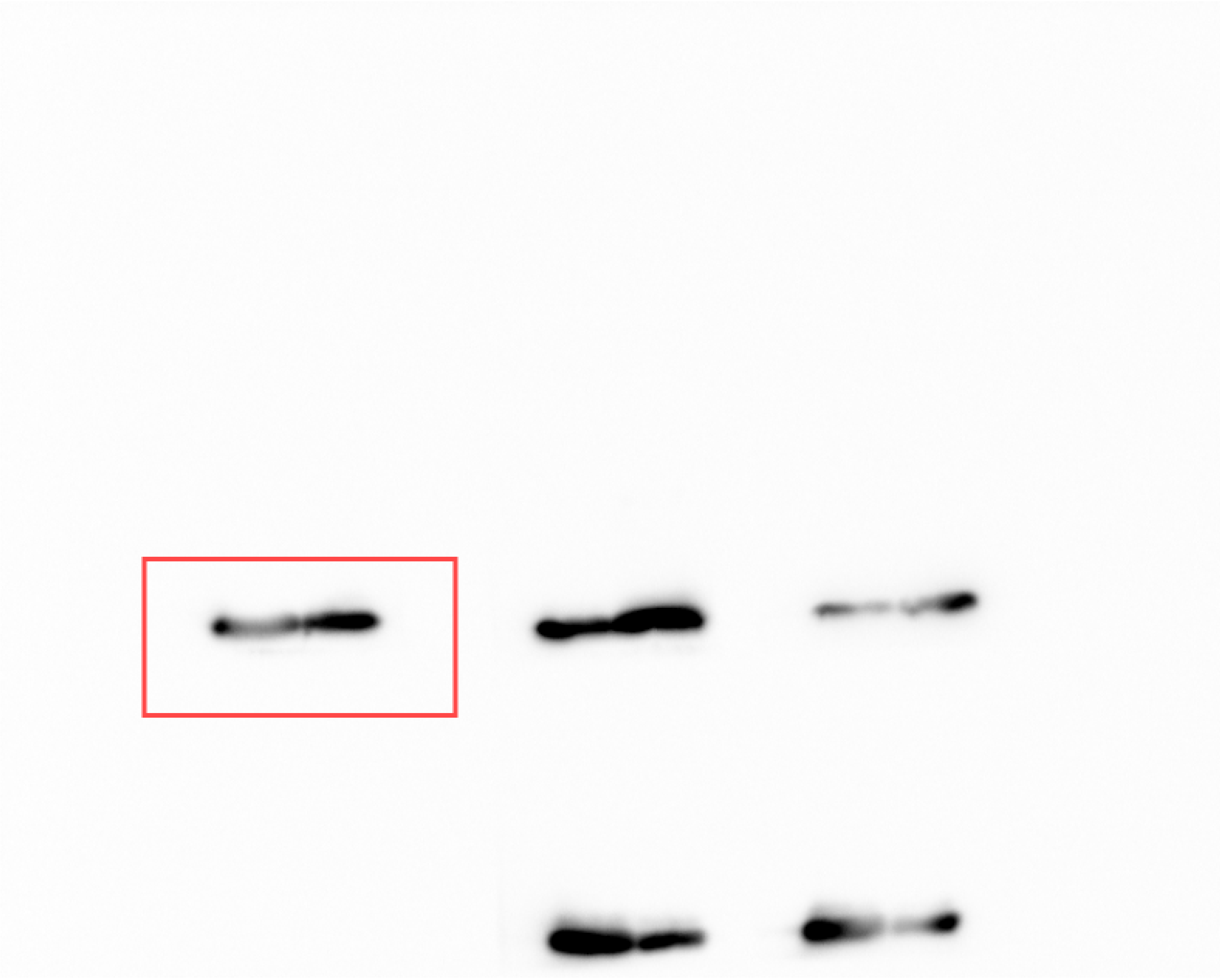

Figure 4G-GAPDH

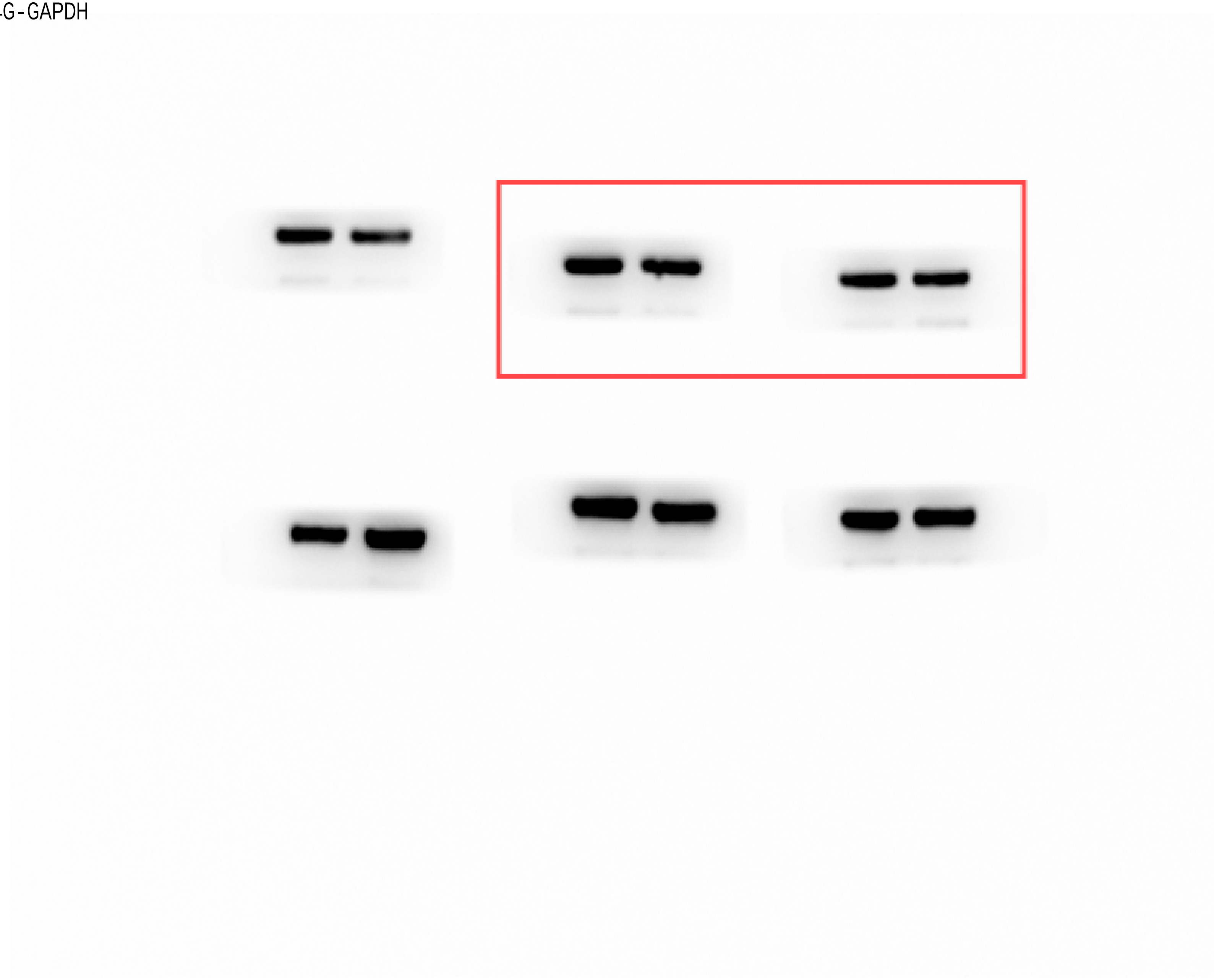

Figure 4H-HMGA2-1

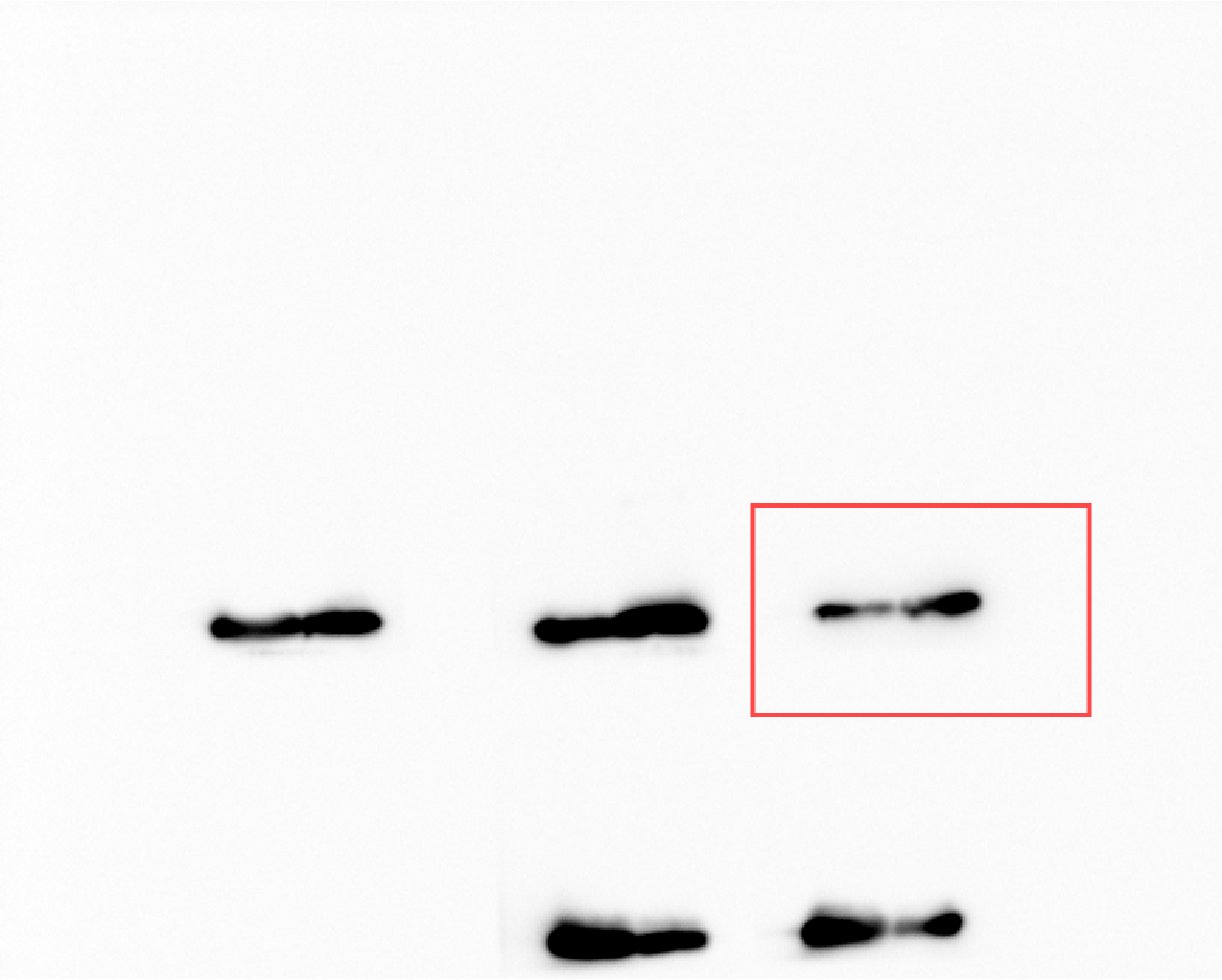

Figure 4H-HMGA2-2

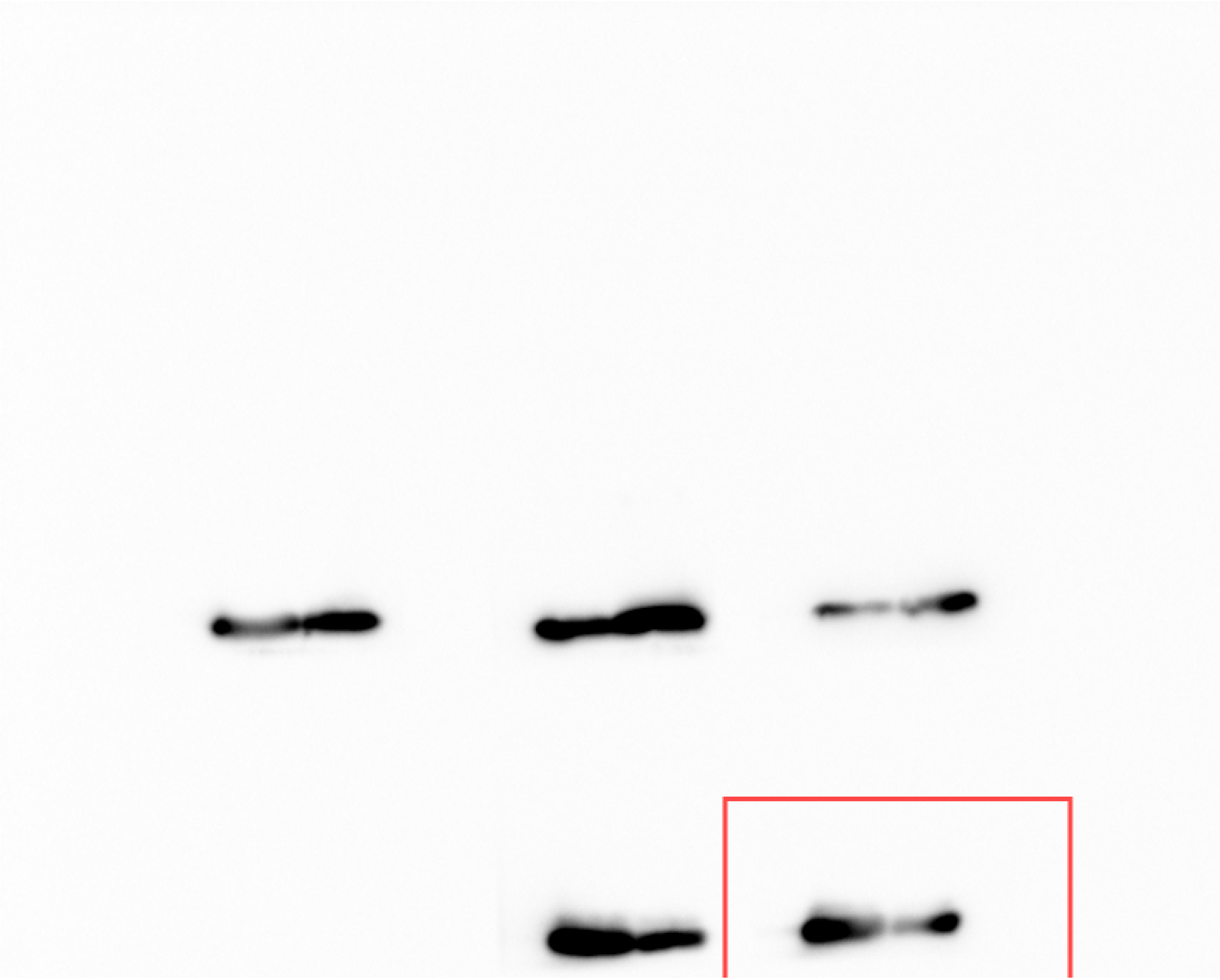

Figure 4H-CCL2-1

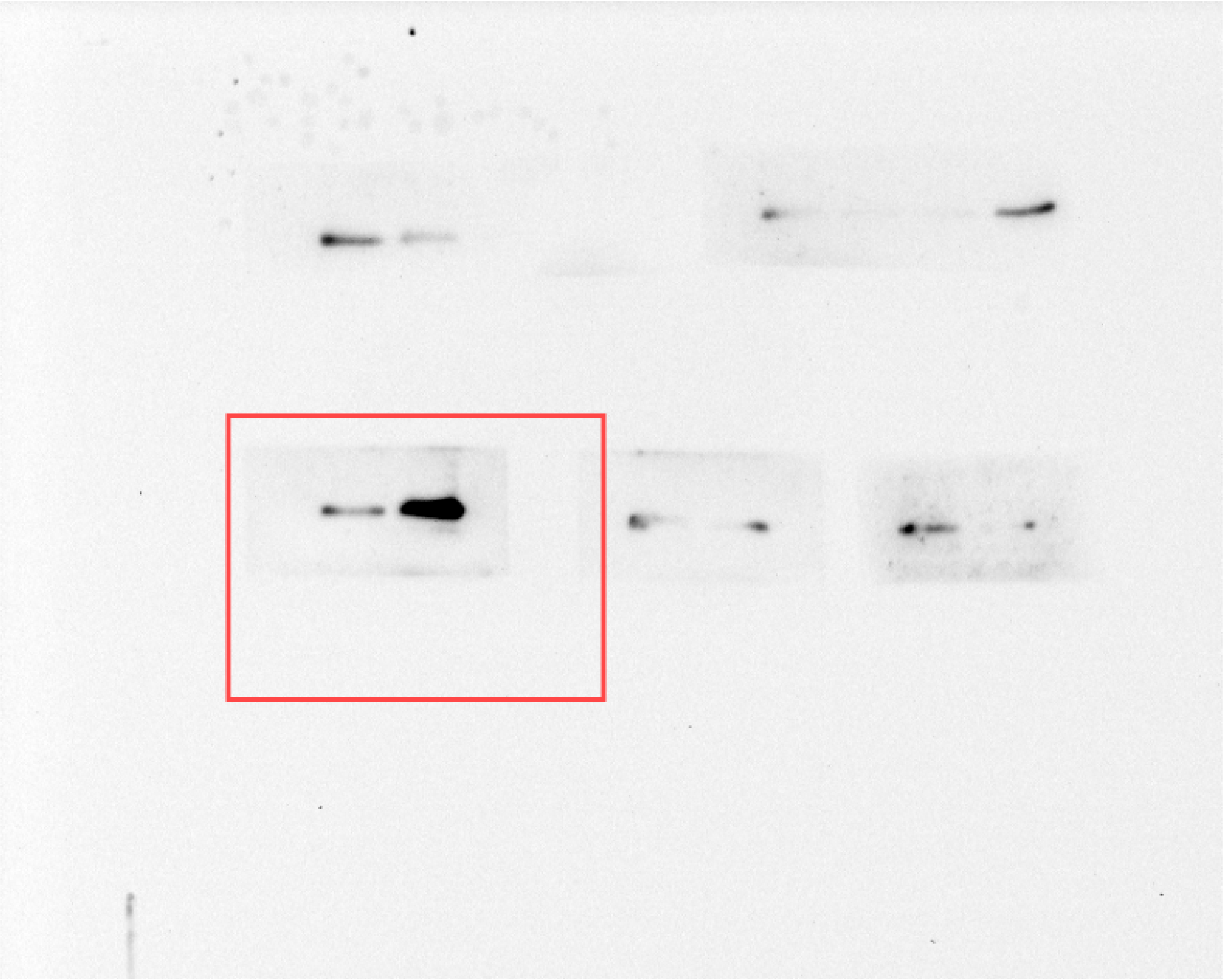

Figure 4H-CCL2-2

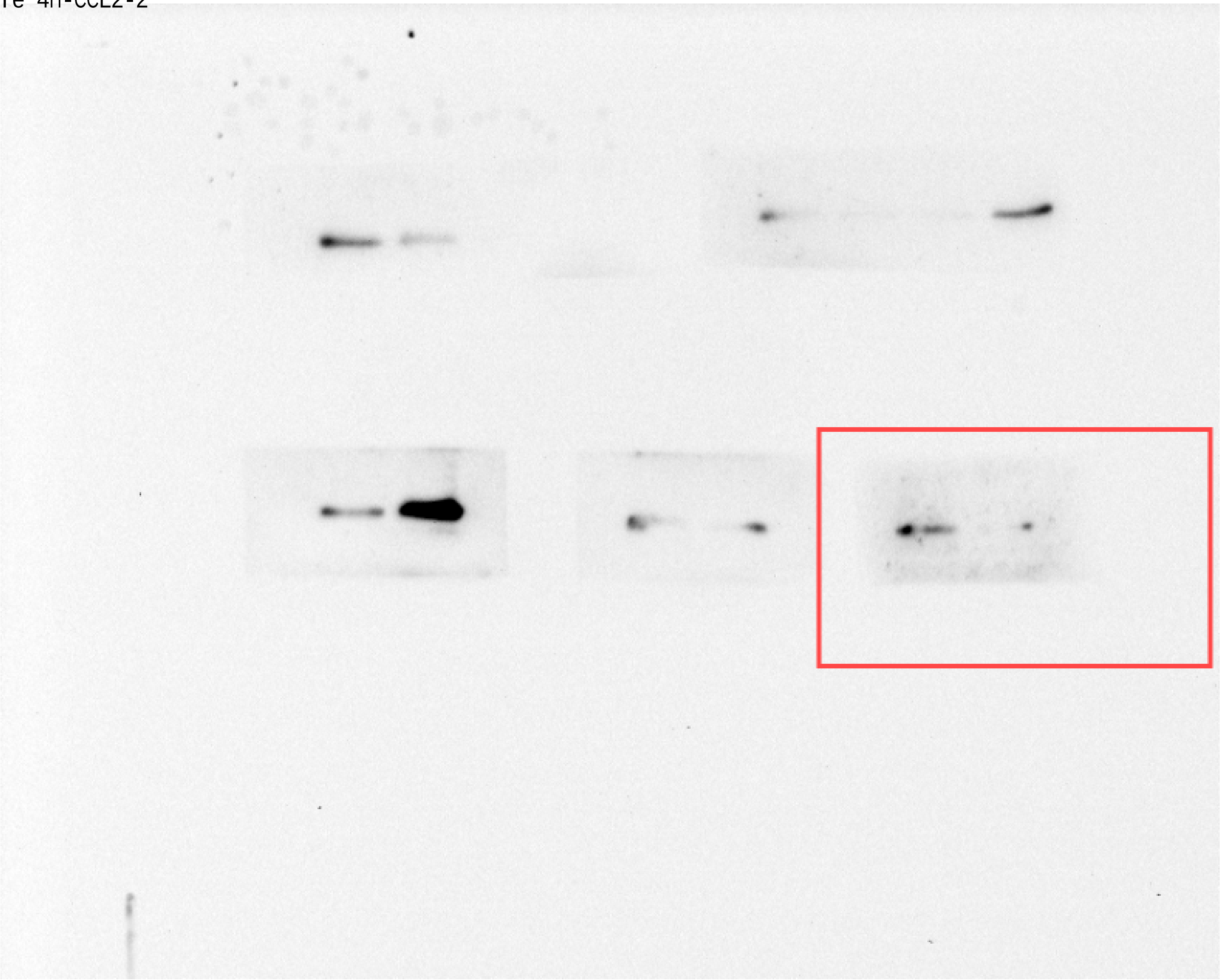

Figure 4H-GAPDH

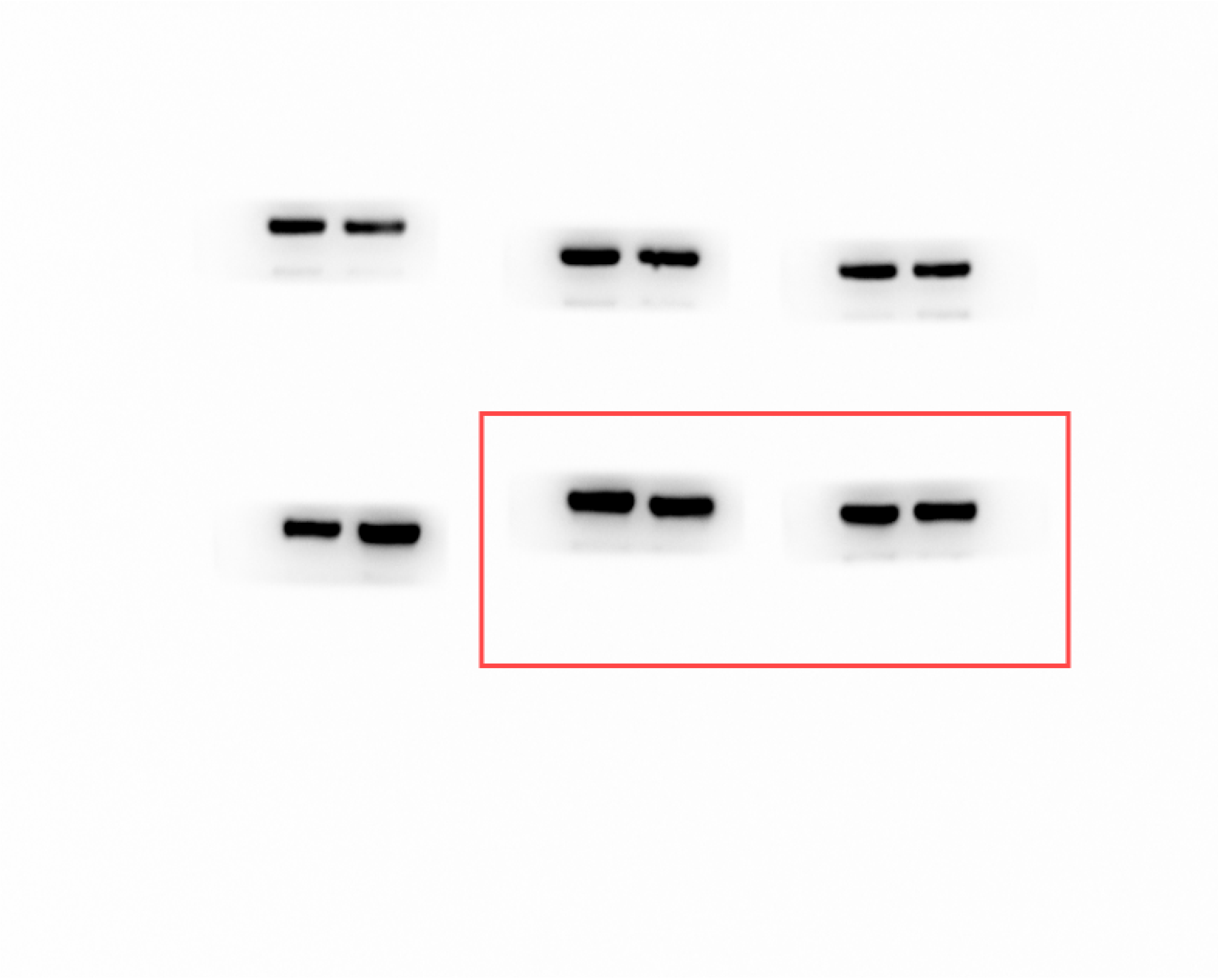

Figure 4K-CCL2

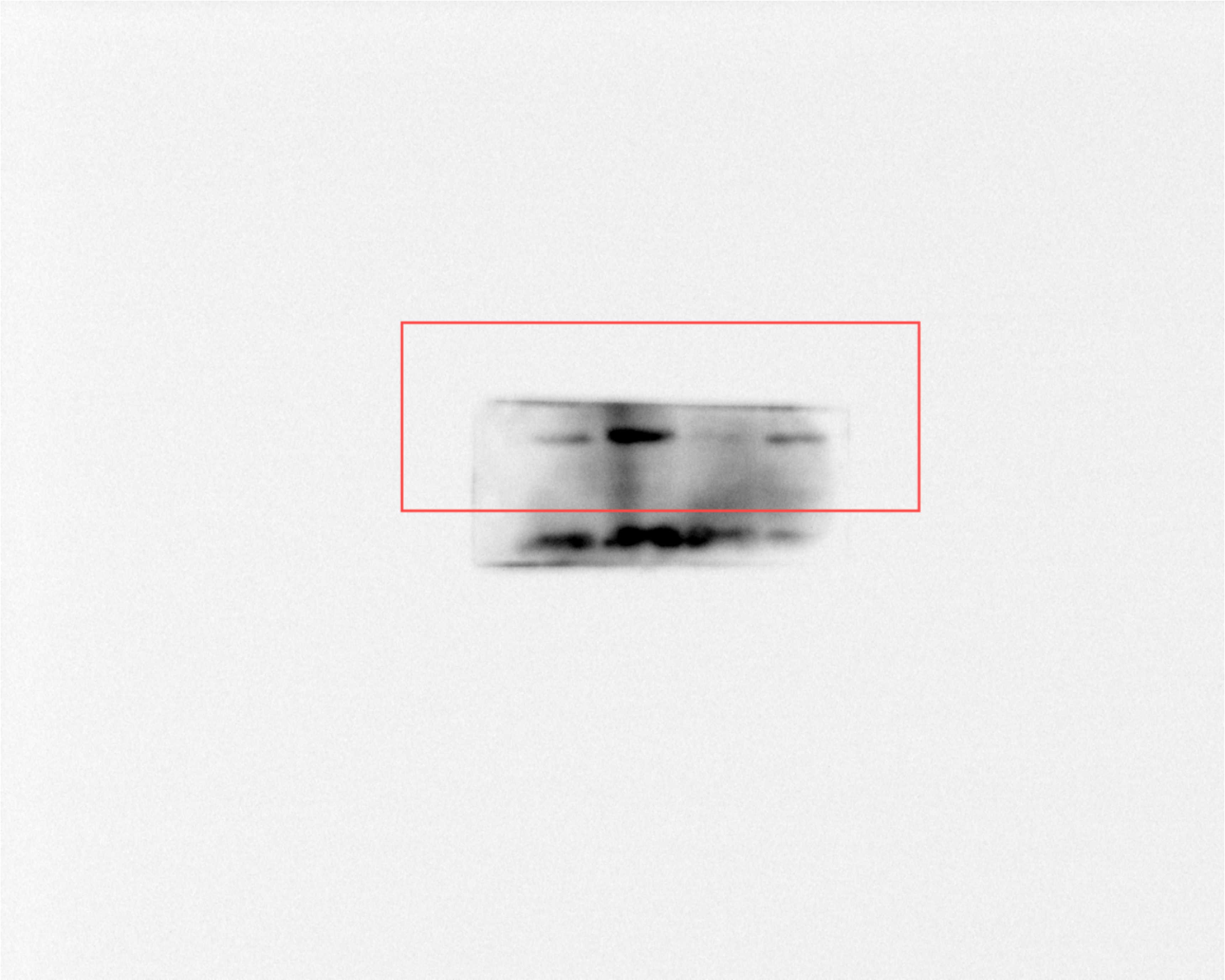

Figure 4K-GAPDH

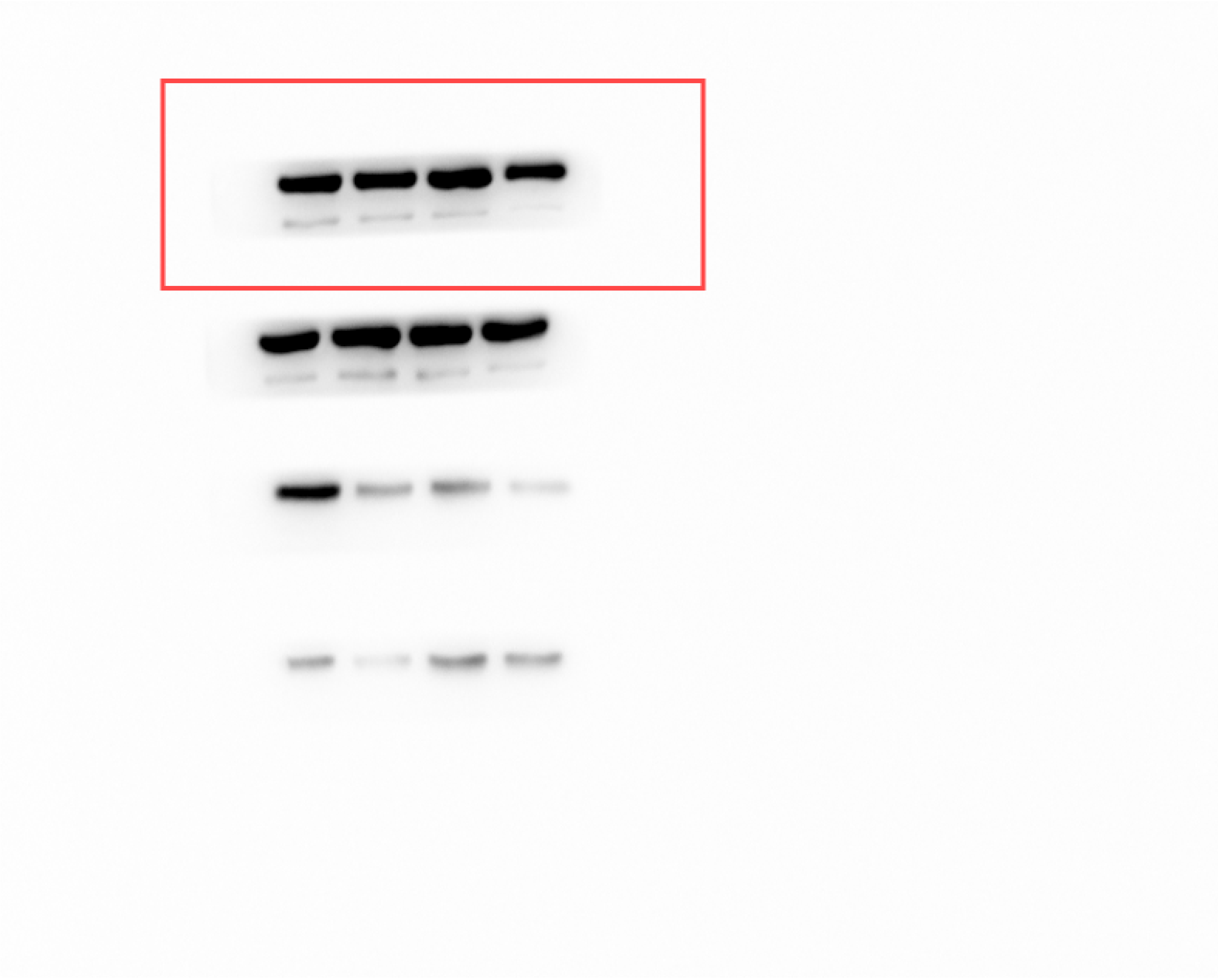

Figure 4L-CCL2

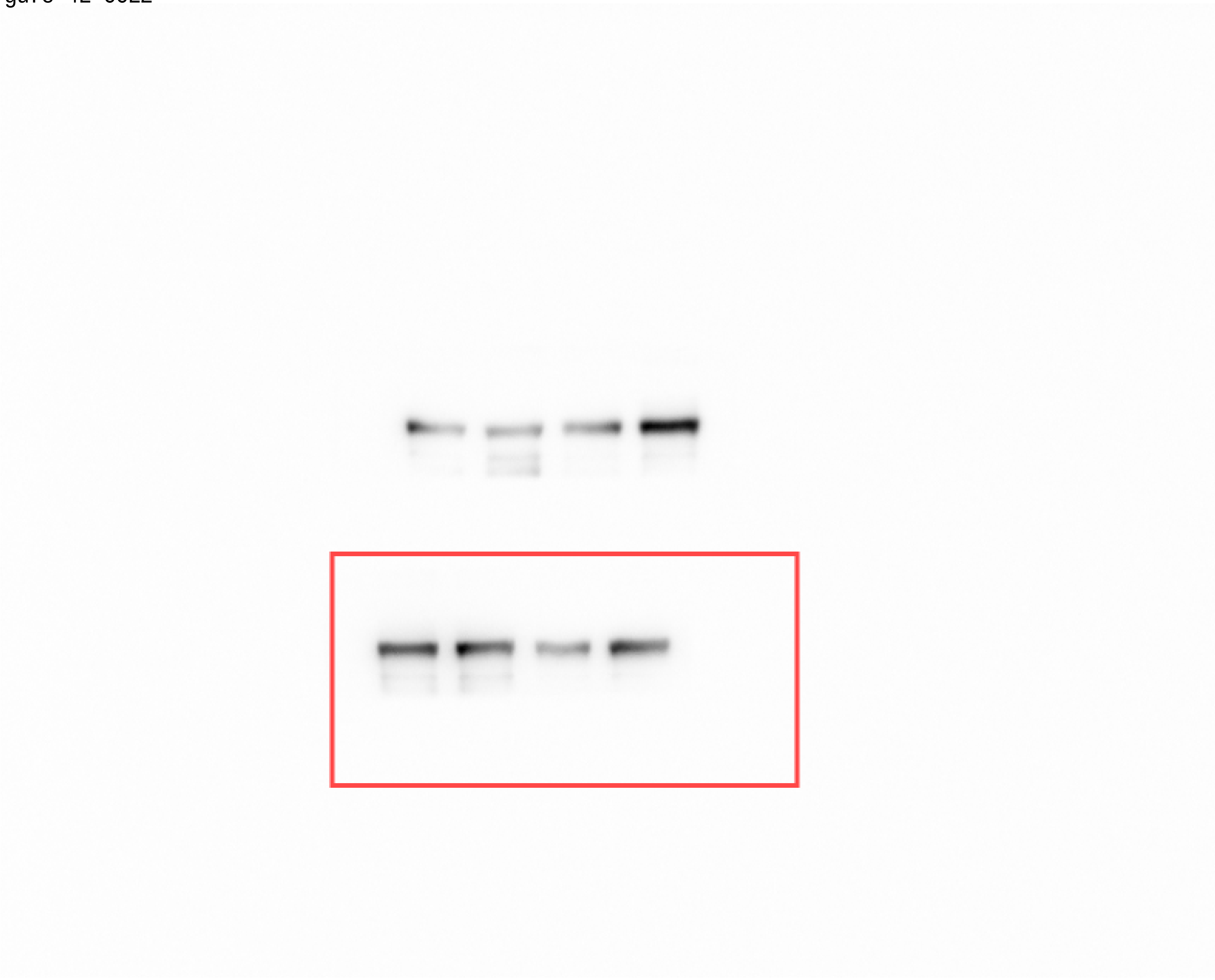

Figure 4L-GAPDH

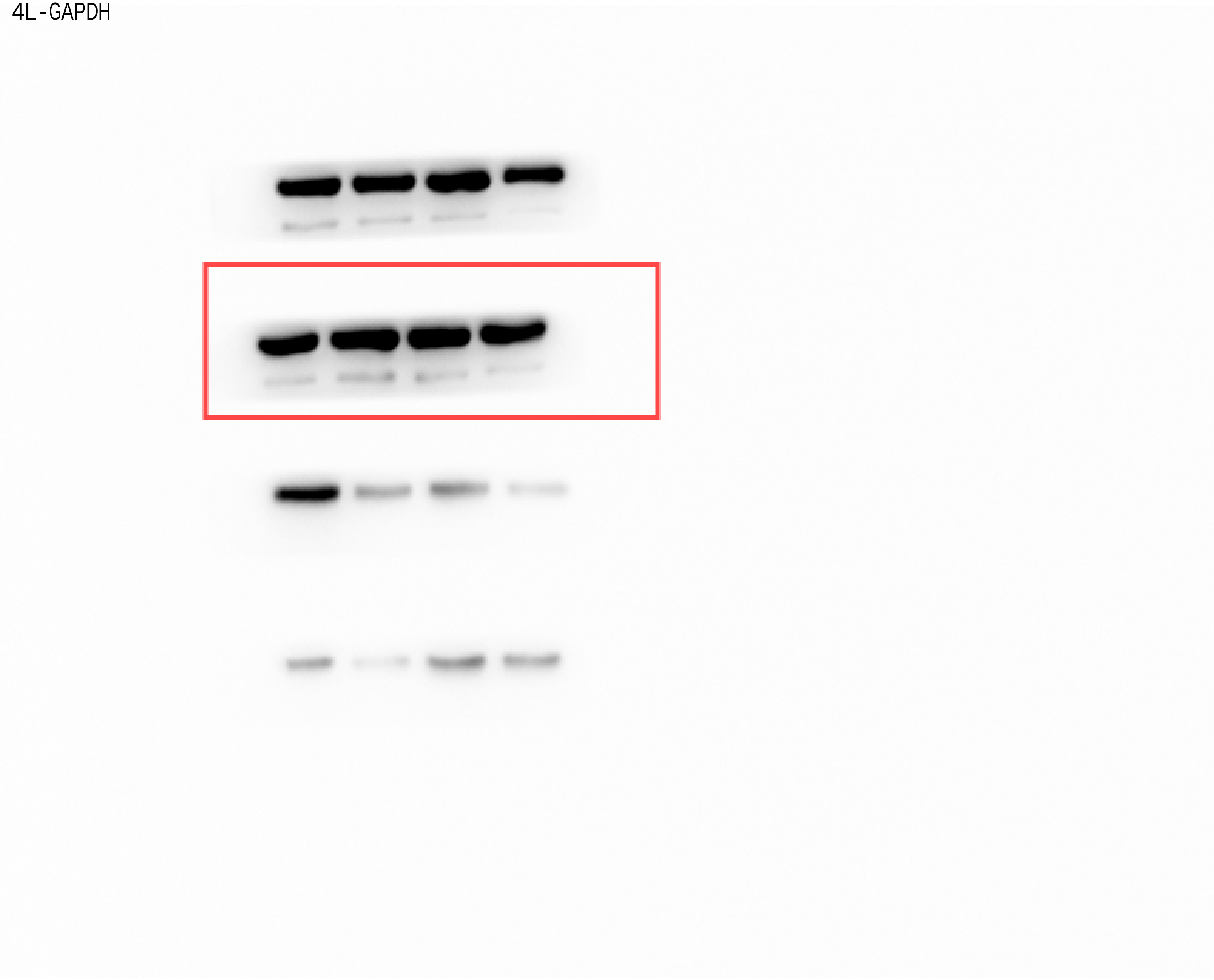

Supplementary Figure 1C-Fra-1

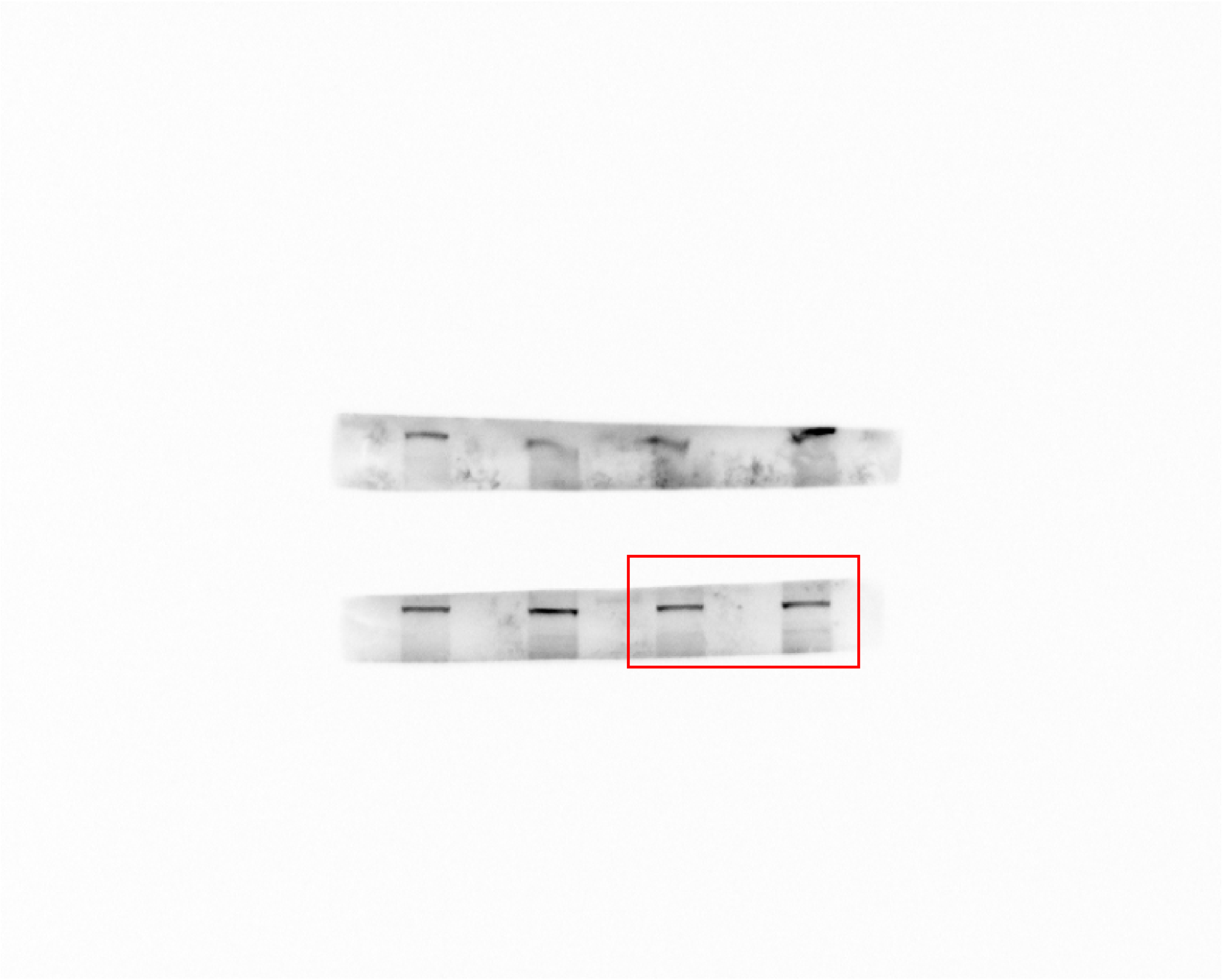

Supplementary Figure 1C-HMGA2

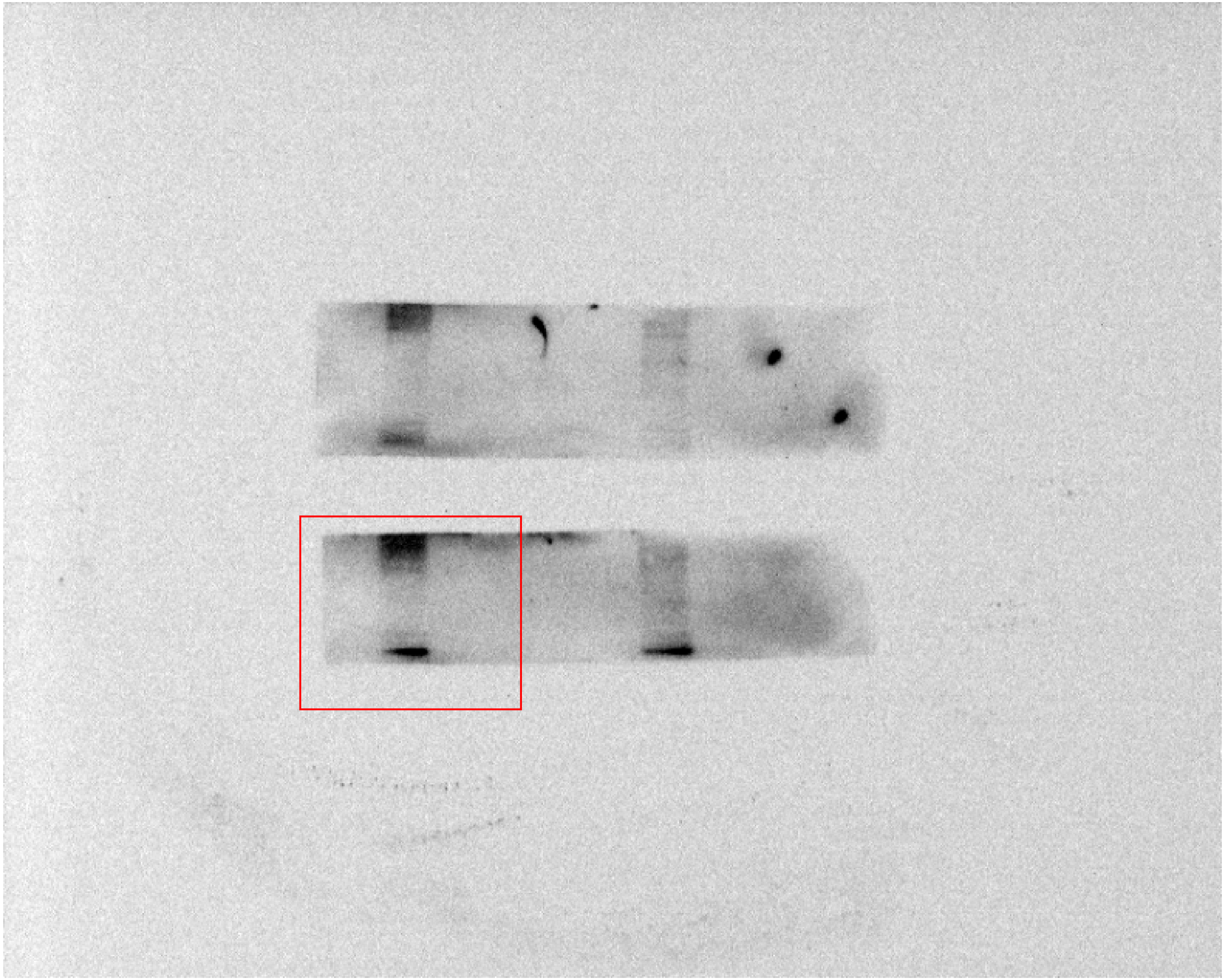

Supplementary Figure 1D-Fra-1

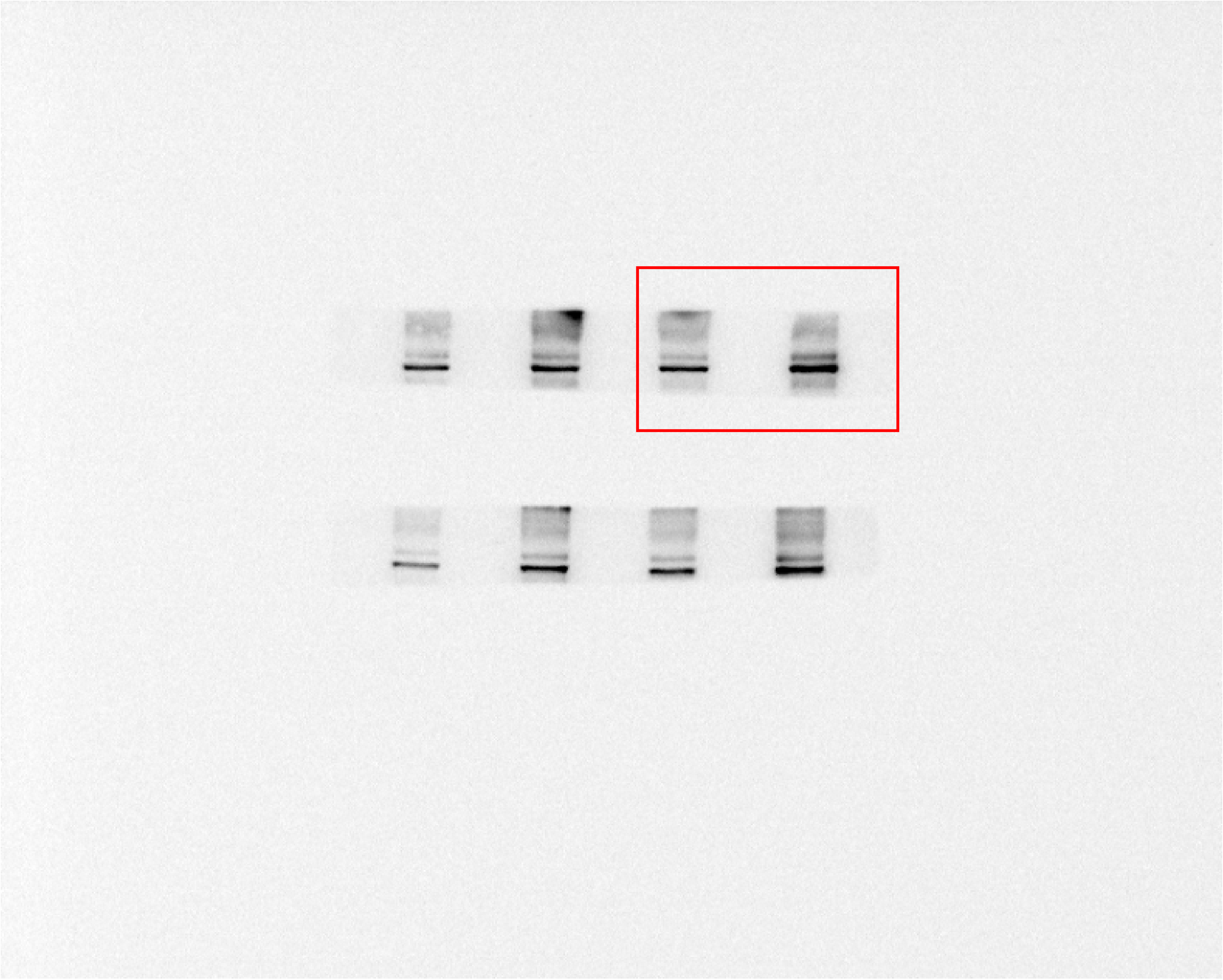

Supplementary Figure 1D-HMGA2

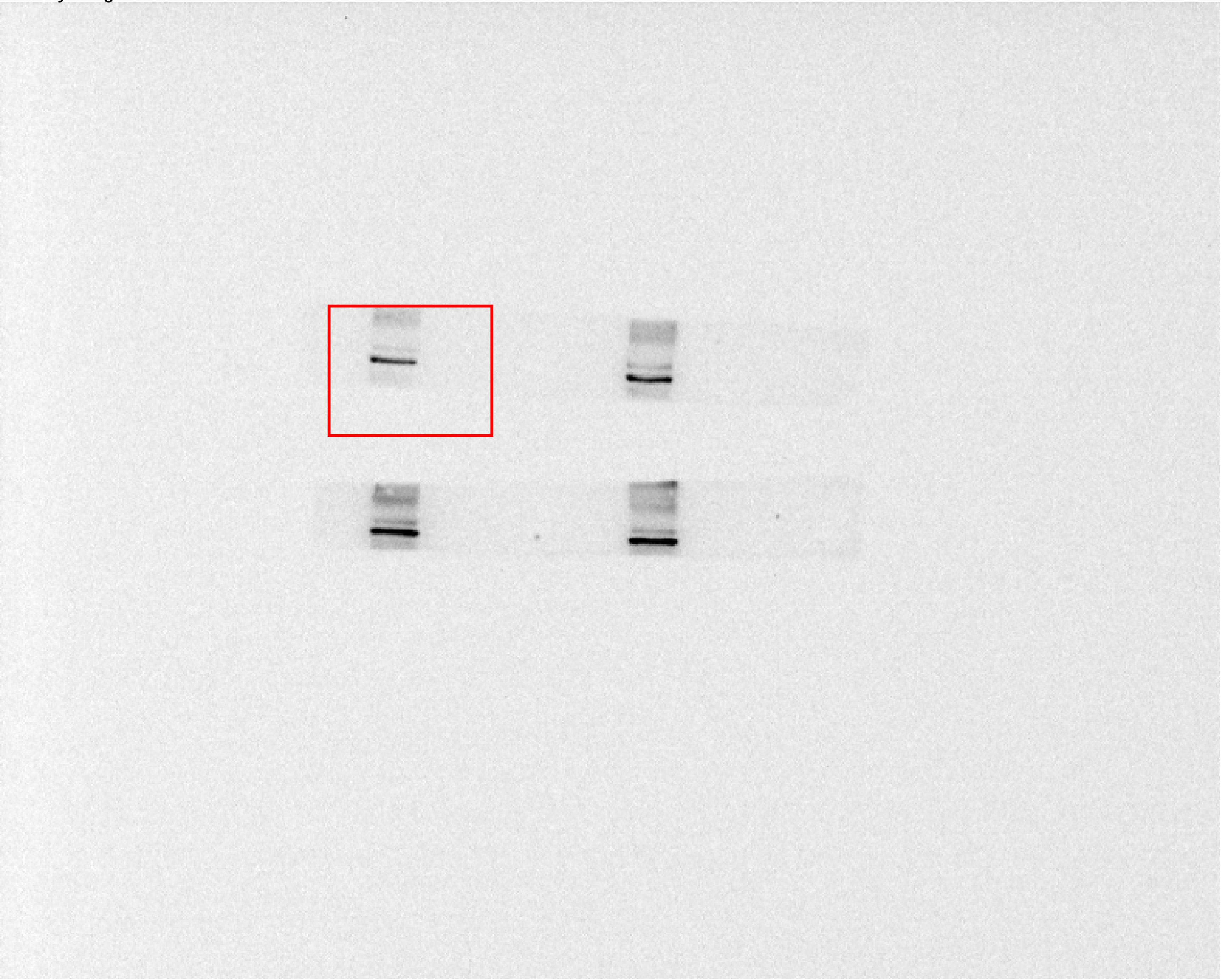

Supplement: Supplementary file 10 — Original Western Blots [file 41420_2025_2724_MOESM10_ESM.pdf]
